# The role of reproductive loss on flock performance: a comparison of nine industry flocks

**DOI:** 10.1093/tas/txab013

**Published:** 2021-01-28

**Authors:** Paul R Shorten, Sara J Edwards, Jenny L Juengel

**Affiliations:** 1 AgResearch Limited, Ruakura Research Centre, Private Bag 3123, Hamilton, New Zealand; 2 AgResearch Limited, Invermay Research Centre, Puddle Alley, Private Bag 50034, Mosgiel, New Zealand

**Keywords:** embryo survival, lamb growth rate, lamb survival, ovulation rate, reproduction, sheep

## Abstract

The reproductive performance of a sheep flock is dependent on a multitude of complex interacting factors. Attaining optimal flock performance requires information about how the reproductive steps are linked and relate to readily available measurements of the state of the flock. The goal was to use data from nine commercial flocks (greater than 300,000 records) to investigate and model the key reproductive steps affecting flock reproductive performance. We also developed a maximum-likelihood based methodology to predict flock ovulation rate based on measurements of the number of fetuses at mid-pregnancy (detected by ultrasound-scanning). The model was used to determine how changes in premating liveweight, age, predicted ovulation rate, number of fetuses at mid-pregnancy, lamb survival and lamb growth rate affect the total lamb liveweight at weaning per ewe exposed to the ram in each flock. The data from the commercial flocks were also used to investigate the role of ewe age and premating liveweight on each reproductive step. Sensitivity analyses were conducted to identify the key reproductive steps affecting flock reproductive performance, with a focus on understanding how these steps vary between flocks. The elasticity for embryo survival was 60% of that for lamb survival for these flocks and the elasticities for ovulation rate were highly variable between flocks (0.16 to 0.50 for mature ewes). This indicates that ovulation rate was near-optimal for some flocks, whereas there was potential to significantly improve flock performance in suboptimal flocks. The elasticity for ewe premating liveweight was highly variable between flocks (−0.03 to 0.84 for mature ewes and −0.18 to 1.39 for ewe lambs), indicating that premating liveweight ranged from optimal to suboptimal between flocks. For these suboptimal farms, the opportunity exists to increase flock performance through improved management of ewe premating liveweight. Reproductive loss was significantly greater in ewe lambs than mature ewes, although the difference is dependent on the stage of reproduction and flock. Predicted ovulation rate was 25% lower for ewe lambs and there was a 30% relative decrease in the predicted embryo survival probability from ovulation to scanning for ewe lambs. There was a 10% relative decrease in lamb survival probability from birth to weaning for ewe lambs and lamb growth rate was 25% lower for ewe lambs.

## INTRODUCTION

A key driver of sheep farm production is the reproductive performance of the flock (Young et al., 2014). The reproductive process requires minimal losses between ovulation rate at mating, mid-pregnancy ultrasound scanning of the number of fetuses, and the number of lambs at weaning, as well as suitable growth of lambs to weaning. Information such as ewe age and premating liveweight can be used to predict the reproductive performance of ewes and the survival and growth of their lambs (Cumming, 1977; Hanrahan, 1982). There are extensive information and associated models about the relationships between ewe state and components of the reproductive pathway (Oldham et al., 2011; Behrendt et al., 2019), however, aspects of these relationships are variable between research flocks (Hanrahan, 1980) and likely to be more variable between commercial farms. These relationships have been assembled into bioeconomic models that relate farm environmental, economic and management variables to the reproduction pathway, which generate important information at a regional scale for a generic average farm (Amer et al., 1999; Morel and Kenyon 2006; Young et al., 2011, 2014; Wall et al., 2018).

We have previously calculated flock elasticities that describe the relative importance of the effect of average premating ewe liveweight (0.81), average ovulation rate (0.33), variance in ovulation rate (−0.095), embryo survival (0.72), lamb survival (1.03), conception failure (0.35), and average ewe age (0.056) on the total kilograms of lamb liveweight at weaning per ewe exposed to the ram in a research flock that had undergone selection for fecundity (Shorten et al., 2020). This indicated that lamb survival had the largest elasticity and that a 1% increase in lamb survival is expected to have a 1.03% increase in the total kilograms of lamb liveweight at weaning per ewe exposed to the ram in this flock. However, there are risks in over extrapolating such models to specific farms due to unique farm conditions. Improved flock specific characterization of the reproductive process will allow for better evaluation of the trade-offs between the reproductive steps for that flock and identification of key targets to improve the reproductive performance of a specific flock, which are likely different from a generic average flock (Morel and Kenyon 2006). Improvements in the collection of on-farm information will allow these models to be recalibrated to individual farms using a subset of traits and recalibration procedures developed using research flocks. Here we demonstrate the feasibility of this approach by developing procedures to estimate ovulation rate and embryo survival from ultrasound information on the number of scanned fetuses.

Thus, the first objective of this study was to investigate how ewe age and premating liveweight affect ovulation rate, number of fetuses at mid-pregnancy, lambing day, lamb survival to weaning and weaning weight in nine New Zealand commercial flocks. The second objective was to assess the role of different parameters on the total weight of lambs weaned per ewe exposed to the ram for this flock. The third objective was to investigate the differences in reproductive performance between mature ewes and ewe lambs. Our primary hypothesis is that there are large between flock differences in reproductive performance and that targets to improve reproductive performance are flock specific.

## MATERIALS AND METHODS

### Experimental Data

The data consisted of more than 300,000 records from nine New Zealand industry flocks collected from 1986 to 2017. Records were extracted with permission from each of the nine flock owners from the New Zealand Sheep Improvement Limited national performance recording database. Each flock size was typically 1,000 ewes. Liveweight and body condition score (BCS) were measured premating, although liveweight measurements were more abundant than BCS measurements. Furthermore, BCS was measured at ultrasound scanning and weaning (liveweight was not measured at scanning or weaning). Ewes were exposed to a ram as a ewe lamb in all flocks, although body condition score measurements were not obtained for ewe lambs.

All ewes were mated under commercial conditions and the rams remained with the ewes for approximately two reproductive cycles (34 d). Ewes were ultrasound-scanned during mid-pregnancy to determine the number of fetuses present. The number of lambs born was not accurately recorded in all years and all flocks. Losses from scanning to lambing are likely 1% to 2% (Willingham et al., 1986; Shorten et al., 2020), and therefore the number of fetuses scanned is a proxy measure of the number of lambs born. The day of lamb birth was based on fetal number and size information obtained at ultrasound scanning (Sergeev et al., 1990; Noia et al., 2002). Lamb weaning weight was also recorded together with the number of lambs weaned per ewe and the predicted lamb age (days) at weaning.

In general, ewes were culled for age after five lambings, although poor performing (low body condition, udder damage, nonpregnant ewes, etc.) ewes were culled at any age to maintain a high-performing flock with a relatively constant size. Animals were managed and observed under standard commercial conditions and all animals had unrestricted access to pasture and water to meet their metabolic requirements.

### Mathematical Models of Embryo Survival, Lamb Survival and Lamb Growth Rate

The model components are outlined in the following sections, which are based on the relationships used to investigate the reproductive performance of a research flock (Shorten et al., 2020). The reproductive performance of ewe lambs is lower than that of mature ewes (Edwards and Juengel, 2017) and therefore separate models were considered for ewe lambs and mature ewes. Ewe age (*A*) terms are omitted from each equation for the ewe lamb models. Ewe lamb premating liveweight was based on liveweight at 8 mo of age, which is the age that ewe lambs were joined with fertile rams.

#### Premating liveweight and ewe age distribution.

Ewe premating liveweight within each age at lambing class *i* (for ages 2 to 9) is described by a normal distribution with mean (*µ*_W,*i*_) and standard deviation (*σ*_W,*i*_). The notation $N(\mu ,\sigma^{2})$ denotes the normal distribution with mean μ and variance $\sigma^{2}$. Ewe age distribution is modeled by the proportion of ewes in the flock of age *i* ($a_{i}$).

#### Ovulation rate and embryo survival.

The distribution of ovulation rate can be described by a lognormal distribution ($LN\left(n;\bar{n},\sigma_{n}^{2}\right)$). Embryo survival to scanning can be described by a binomial process (Restall and Griffiths, 1976) with a survival probability that is dependent on the ovulation rate (Geisler et al. 1977). The mean number of ova ($\bar{n}$) is typically lower at age 2, increases from age 2 to 5 (Schoenian and Burfening 1990) and decreases thereafter and can be described by a quadratic function:

$$\begin{array}{l} \bar{n}=\beta_{n0}+\beta_{n1}A+\beta_{n2}A^{2}+\beta_{w1}W+\beta_{w2}W^{2}, \end{array}$$ (1)

with a similar quadratic effect of weight (Morley et al., 1978) (see Table 1 for descriptions and values of parameters). The effect of ewe age on the mean ovulation rate adjusted for premating liveweight is given by Eq. (1) with $W=\bar{W}$ (where $\bar{W}$ denotes the average liveweight of all ewes). The number of scanned fetuses is therefore described by a compound lognormal-binomial distribution where the probability of observing *k* scanned fetuses is

**Table 1.** Table of model parameters, values and descriptions for mature ewes

Parameter	Description	All flocks^*a*,*b*,*c*^	Flock 1	Flock 2	Flock 3	Flock 4	Flock 5	Flock 6	Flock 7	Flock 8	Flock 9
$\beta_{n0}$	Intercept term for the relationship between age and weight on ovulation rate, ova	0.89 ± 0.021	−1.59 ± 0.041	0.78 ± 0.16	−0.26 ± 0.092	0.48 ± 0.21	0.29 ± 0.30	−0.25 ± 1.08	0.038 ± 0.075	1.63 ± 0.14	0.47 ± 0.069
$\beta_{n1}$	Linear effect of age on ovulation rate, ova yr^−1^	0.30 ± 0.017	0.25 ± 0.024	0.47 ± 0.073	0.22 ± 0.054	0.18 ± 0.075	0.14 ± 0.064	0.42 ± 0.08	0.27 ± 0.044	0.13 ± 0.036	0.23 ± 0.037
$\beta_{n2}$	Quadratic effect of age on ovulation rate, ova yr^−2^	−0.025 ± 0.00223	−0.022 ± 0.0032	−0.044 ± 0.0093	−0.015 ± 0.0078	−0.0078 ± 0.0092	−0.017 ± 0.0087	−0.042 ± 0.010	−0.024 ± 0.0053	−0.0095 ± 0.0049	−0.023 ± 0.0045
$\beta_{w1}$	Linear effect of premating liveweight on ovulation rate, ova kg^−1^	0.026 ± 0.00064	0.097 ± 0.017	−0.0041 ± 0.0054	0.029 ± 0.0034	0.024 ± 0.0071	0.045 ± 0.0075	0.049 ± 0.028	0.019 ± 0.0025	0.011 ± 0.0054	0.030 ± 0.0033
$\beta_{w2}$	Quadratic effect of premating liveweight on ovulation rate, ova kg^−2^	−0.00015 ± 0.0000066	−0.00058 ± 0.000019	0.00011 ± 0.000040	−0.000068 ± 0.000024	−0.00013 ± 0.000057	−0.00025 ± 0.000051	−0.00022 ± 0.00018	0.000039 ± 0.000020	−0.000012 ± 0.000052	−0.000078 ± 0.000031
$\sigma_{n}$	Standard deviation in ovulation rate, ova	0.57 ± 0.0050	0.48 ± 0.0068	0.43 ± 0.012	0.45 ± 0.0087	0.46 ± 0.013	0.38 ± 0.033	0.71 ± 0.021	0.45 ± 0.0082	0.36 ± 0.017	0.59 ± 0.010
$p_{n}$	Probability of embryo survival to scanning for single ovulation	0.92 ± 0.0012	0.94 ± 0.0023	0.89 ± 0.0047	0.92 ± 0.0037	0.88 ± 0.0061	0.95 ± 0.0052	0.99 ± 0.004	0.91 ± 0.0033	0.97 ± 0.0038	0.97 ± 0.0027
µ _W,2_	Mean premating liveweight for 2-yr-old ewe, kg	61.43 ± 0.05	63.0 ± 0.09	67.5 ± 0.15	66.2 ± 0.13	63.36 ± 0.21	66.65 ± 0.23	69.51 ± 0.15	64.75 ± 0.10	52.32 ± 0.10	54.84 ± 0.07
σ _W,2_	Standard deviation in premating liveweight for 2-yr-old ewe, kg	8.89 ± 0.03	8.61 ± 0.06	7.21 ± 0.11	8.12 ± 0.10	7.58 ± 0.14	8.87 ± 0.16	7.18 ± 0.11	6.76 ± 0.09	5.60 ± 0.07	5.59 ± 0.05
µ _W,3_	Mean premating liveweight for 3-yr-old ewe, kg	66.43 ± 0.06	65.55 ± 0.10	73.8 ± 0.18	72.31 ± 0.18	69.61 ± 0.24	75.25 ± 0.28	73.63 ± 0.20	70.43 ± 0.13	56.93 ± 0.12	61.58 ± 0.10
σ _W,3_	Standard deviation in premating liveweight for 3-yr-old ewe, kg	9.05 ± 0.04	7.71 ± 0.07	6.98 ± 0.14	8.61 ± 0.13	7.28 ± 0.22	8.34 ± 0.19	8.09 ± 0.15	7.37 ± 0.10	5.76 ± 0.09	5.96 ± 0.07
µ _W,4_	Mean premating liveweight for 4 yr old ewe, kg	66.70 ± 0.07	66.60 ± 0.13	75.76 ± 0.24	77.27 ± 0.19	72.58 ± 0.28	77.90 ± 0.40	78.28 ± 0.26	74.22 ± 0.16	60.10 ± 0.16	65.18 ± 0.13
σ _W,4_	Standard deviation in premating liveweight for 4-yr-old ewe, kg	9.46 ± 0.05	7.52 ± 0.09	7.60 ± 0.24	8.56 ± 0.14	7.53 ± 0.28	8.30 ± 0.28	8.92 ± 0.18	7.82 ± 0.11	5.80 ± 0.11	6.30 ± 0.10
µ _W,5_	Mean premating liveweight for 5-yr-old ewe, kg	70.96 ± 0.09	68.1 ± 7.35	76.58 ± 0.29	77.26 ± 0.26	73.87 ± 0.34	79.86 ± 0.60	79.84 ± 0.37	75.18 ± 0.20	61.33 ± 0.23	66.84 ± 0.19
σ _W,5_	Standard deviation in premating liveweight for 5-yr-old ewe, kg	9.25 ± 0.07	0.15 ± 0.11	7.74 ± 0.22	9.04 ± 0.17	6.89 ± 0.28	8.12 ± 0.46	8.62 ± 0.26	7.93 ± 0.16	6.29 ± 0.18	6.87 ± 0.14
µ _W,6_	Mean premating liveweight for 6-yr-old ewe, kg	70.36 ± 0.13	68.5 ± 0.20	76.24 ± 0.38	83.47 ± 1.35	73.9 ± 0.54	82.03 ± 1.22	79.70 ± 0.61	74.34 ± 0.32	60.44 ± 0.38	66.56 ± 0.27
σ _W,6_	Standard deviation in premating liveweight for 6-yr-old ewe, kg	8.94 ± 0.11	7.08 ± 0.13	7.29 ± 0.27	11.21 ± 0.82	7.90 ± 0.48	9.93 ± 1.09	9.59 ± 0.46	8.02 ± 0.25	6.27 ± 0.34	6.71 ± 0.20
µ _W,7_	Mean premating liveweight for 7-yr-old ewe, kg	68.78 ± 0.23	67.37 ± 0.36	77.27 ± 1.13	79.38 ± 0.11	71.98 ± 1.33	84.59 ± 1.99	77.82 ± 0.99	74.58 ± 0.59	61.92 ± 0.83	66.75 ± 0.46
σ _W,7_	Standard deviation in premating liveweight for 7-yr-old ewe, kg	8.43 ± 0.18	7.44 ± 0.24	6.97 ± 0.78	0.18 ± 0.12	7.77 ± 0.80	5.97 ± 1.78	8.25 ± 0.58	8.22 ± 0.49	6.27 ± 0.48	6.43 ± 0.32
µ _W,8_	Mean premating liveweight for 8-yr-old ewe, kg	69.0 ± 0.70	NA	76.93 ± 3.51	NA	70.92 ± 1.94	NA	NA	73.67 ± 1.30	63.86 ± 1.86	67.56 ± 1.20
σ _W,8_	Standard deviation in premating liveweight for 8-yr-old ewe, kg	7.7 ± 0.78	NA	7.61 ± 2.08	NA	4.12 ± 1.63	NA	NA	7.98 ± 1.57	3.89 ± 1.20	4.08 ± 0.70
µ _W,9_	Mean premating liveweight for 9-yr-old ewe, kg	69.25 ± 1.76	NA	NA	NA	NA	NA	NA	72.61 ± 3.67	NA	NA
σ _W,9_	Standard deviation in premating liveweight for 9-yr-old ewe, kg	5.69 ± 1.39	NA	NA	NA	NA	NA	NA	5.19 ± 3.39	NA	NA
$a_{2}$	Proportion of ewes in the flock of age 2	0.392	0.357	0.404	0.394	0.356	0.493	0.472	0.362	0.434	0.427
$a_{3}$	Proportion of ewes in the flock of age 3	0.269	0.263	0.280	0.279	0.311	0.284	0.275	0.260	0.277	0.264
$a_{4}$	Proportion of ewes in the flock of age 4	0.174	0.179	0.165	0.186	0.200	0.140	0.152	0.176	0.168	0.161
$a_{5}$	Proportion of ewes in the flock of age 5	0.097	0.104	0.087	0.104	0.084	0.060	0.066	0.114	0.084	0.089
$a_{6}$	Proportion of ewes in the flock of age 6	0.047	0.059	0.051	0.027	0.039	0.020	0.027	0.059	0.030	0.043
$a_{7}$	Proportion of ewes in the flock of age 7	0.017	0.028	0.010	0.0071	0.0091	0.0029	0.0074	0.021	0.0067	0.014
$a_{8}$	Proportion of ewes in the flock of age 8	0.0048	0.0096	0.0020	0.0029	0.00059	0	0.00062	0.0058	0.00055	0.00089
$a_{9}$	Proportion of ewes in the flock of age 9	0.00042	0.00037	0.00038	0.00021	0.000066	0	0	0.0017	0	0
$\beta_{10}$	Intercept term for the relationship between age and weight and lamb survival for singles	1.81 ± 0.87	2.57 ± 2.15	−3.42 ± 3.16	−1.81 ± 2.43	−3.74 ± 3.15	1.84 ± 6.49	5.46 ± 8.21	1.02 ± 3.26	−6.46 ± 5.01	−2.89 ± 3.63
$\beta_{W11}$	Linear effect of premating liveweight on lamb survival for singles, kg^−1^	0.00086 ± 0.026	−0.0083 ± 0.068	0.17 ± 0.088	0.10 ± 0.068	0.14 ± 0.093	−0.051 ± 0.18	−0.12 ± 0.22	0.0057 ± 0.096	0.28 ± 0.18	0.15 ± 0.12
$\beta_{W12}$	Quadratic effect of premating liveweight on lamb survival for singles, kg^−2^	−0.0000093 ± 0.00019	0.00011 ± 0.00053	−0.0012 ± 0.00060	−0.00061 ± 0.00048	−0.0010 ± 0.00068	0.00055 ± 0.0014	0.00075 ± 0.0014	0.000020 ± 0.00070	−0.0024 ± 0.0016	−0.0012 ± 0.0010
$\beta_{A11}$	Linear effect of age on lamb survival for singles, yr^−1^	0.16 ± 0.12	−0.43 ± 0.24	−0.34 ± 0.35	−0.31 ± 0.45	0.58 ± 0.39	1.27 ± 1.17	1.11 ± 0.65	0.34 ± 0.31	0.53 ± 0.55	0.32 ± 0.38
$\beta_{A12}$	Quadratic effect of age on lamb survival for singles, yr^−2^	−0.027 ± 0.015	0.042 ± 0.030	0.021 ± 0.044	0.052 ± 0.067	−0.083 ± 0.048	−0.20 ± 0.16	−0.15 ± 0.081	−0.019 ± 0.041	−0.12 ± 0.072	−0.041 ± 0.046
$\beta_{20}$	Intercept term for the relationship between age and weight and lamb survival for twins	0.83 ± 0.29	−2.36 ± 0.75	−1.61 ± 1.52	−3.77 ± 1.24	−9.04 ± 1.62	−0.68 ± 1.83	−0.63 ± 1.86	0.49 ± 1.29	−0.78 ± 1.72	−4.73 ± 1.10
$\beta_{W21}$	Linear effect of premating liveweight on lamb survival for twins, kg^−1^	0.0043 ± 0.0085	0.11 ± 0.024	0.062 ± 0.042	0.10 ± 0.033	0.26 ± 0.047	0.024 ± 0.052	0.062 ± 0.050	−0.0014 ± 0.036	0.070 ± 0.060	0.17 ± 0.037
$\beta_{W22}$	Quadratic effect of premating liveweight on lamb survival for twins, kg^−2^	−0.000092 ± 0.000062	−0.00079 ± 0.00018	−0.00041 ± 0.00029	−0.00064 ± 0.00022	−0.0018 ± 0.00033	−0.00017 ± 0.00036	−0.00050 ± 0.00033	−0.000056 ± 0.00025	−0.00065 ± 0.00053	−0.0015 ± 0.00030
$\beta_{A21}$	Linear effect of age on lamb survival for twins, yr^−1^	0.44 ± 0.038	0.14 ± 0.070	0.29 ± 0.15	0.76 ± 0.16	0.38 ± 0.16	0.89 ± 0.26	0.34 ± 0.16	0.65 ± 0.095	0.41 ± 0.16	0.82 ± 0.12
$\beta_{A22}$	Quadratic effect of age on lamb survival for twins, yr^−2^	−0.058 ± 0.0048	−0.026 ± 0.0089	−0.036 ± 0.020	−0.11 ± 0.022	−0.064 ± 0.021	−0.11 ± 0.036	−0.054 ± 0.021	−0.068 ± 0.012	−0.059 ± 0.022	−0.093 ± 0.014
$\beta_{30}$	Intercept term for the relationship between age and weight and lamb survival for triplets	−0.18 ± 0.36	−3.48 ± 0.86	4.79 ± 4.63	−0.50 ± 3.64	−0.48 ± 5.08	−3.68 ± 3.57	1.51 ± 1.50	−0.13 ± 2.66	−6.58 ± 1.90	−2.43 ± 1.31
$\beta_{W31}$	Linear effect of premating liveweight on lamb survival for triplets, kg^−1^	−0.00035 ± 0.010	0.099 ± 0.025	−0.16 ± 0.12	−0.017 ± 0.092	−0.020 ± 0.14	0.066 ± 0.095	−0.025 ± 0.039	−0.013 ± 0.069	0.21 ± 0.065	0.072 ± 0.043
$\beta_{W32}$	Quadratic effect of premating liveweight on lamb survival for triplets, kg^−2^	−0.000038 ± 0.000075	−0.00078 ± 0.00018	0.00096 ± 0.00078	0.000096 ± 0.00056	0.000065 ± 0.00098	−0.00041 ± 0.00062	0.000032 ± 0.00025	0.00010 ± 0.00040	−0.0018 ± 0.00054	−0.00061 ± 0.00033
$\beta_{A31}$	Linear effect of age on lamb survival for triplets, yr^−1^	0.53 ± 0.042	0.48 ± 0.061	1.06 ± 0.42	0.90 ± 0.39	0.57 ± 0.44	1.02 ± 0.33	0.51 ± 0.13	0.49 ± 0.18	0.61 ± 0.16	0.57 ± 0.13
$\beta_{A32}$	Quadratic effect of age on lamb survival for triplets, yr^−2^	−0.070 ± 0.0052	−0.066 ± 0.0075	−0.13 ± 0.051	−0.11 ± 0.050	−0.063 ± 0.047	−0.13 ± 0.044	−0.061 ± 0.017	−0.054 ± 0.020	−0.080 ± 0.020	−0.067 ± 0.016
$\beta_{40}$	Intercept term for the relationship between age and weight and lamb survival for quadruplets	−5.81 ± 1.78	−14.1 ± 5.0	NA	NA	NA	NA	−2.62 ± 3.51	NA	NA	−5.64 ± 7.44
$\beta_{W41}$	Linear effect of premating liveweight on lamb survival for quadruplets, kg^−1^	0.14 ± 0.048	0.38 ± 0.14	NA	NA	NA	NA	0.065 ± 0.090	NA	NA	0.15 ± 0.23
$\beta_{W42}$	Quadratic effect of premating liveweight on lamb survival for quadruplets, kg^−2^	−0.00096 ± 0.00032	−0.0027 ± 0.0010	NA	NA	NA	NA	−0.00058 ± 0.00057	NA	NA	−0.0011 ± 0.0018
$\beta_{A41}$	Linear effect of age on lamb survival for quadruplets, yr^−1^	0.38 ± 0.18	0.26 ± 0.33	NA	NA	NA	NA	0.80 ± 0.29	NA	NA	0.46 ± 0.48
$\beta_{A42}$	Quadratic effect of age on lamb survival for quadruplets, yr^−2^	−0.056 ± 0.022	−0.036 ± 0.039	NA	NA	NA	NA	−0.10 ± 0.037	NA	NA	−0.056 ± 0.067
$G_{M}$	Growth rate advantage of ram lamb over ewe lamb, kg d^−1^	0.027 ± 0.00034	0.020 ± 0.0066	0.033 ± 0.0015	0.025 ± 0.0012	0.030 ± 0.0019	0.033 ± 0.0014	0.024 ± 0.0010	0.030 ± 0.0011	0.025 ± 0.0010	0.032 ± 0.00058
$\gamma_{1}$	Lamb growth rate for birth rank 1, kg day^−1^	0.18 ± 0.0031	0.27 ± 0.0092	0.29 ± 0.013	0.14 ± 0.0099	0.26 ± 0.015	0.29 ± 0.014	0.25 ± 0.019	0.29 ± 0.010	0.26 ± 0.014	0.27 ± 0.0058
$\theta_{1}$	Effect of premating ewe liveweight on lamb growth rate for birth rank 1, d^−1^	0.0022 ± 0.000047	0.00074 ± 0.00014	0.00086 ± 0.00018	0.0027 ± 0.00014	0.0010 ± 0.00022	0.0011 ± 0.00019	0.0012 ± 0.00026	0.0011 ± 0.00015	0.00046 ± 0.00024	0.00033 ± 0.000095
$\gamma_{2}$	Lamb growth rate for birth rank 2, kg d^−1^	0.14 ± 0.0015	0.17 ± 0.0036	0.23 ± 0.0081	0.13 ± 0.0059	0.23 ± 0.010	0.22 ± 0.0068	0.22 ± 0.0064	0.25 ± 0.061	0.19 ± 0.0056	0.20 ± 0.0029
$\theta_{2}$	Effect of premating ewe liveweight on lamb growth rate for birth rank 2, d^−1^	0.0020 ± 0.000021	0.0013 ± 0.000056	0.0011 ± 0.00011	0.0020 ± 0.000078	0.0010 ± 0.00014	0.00058 ± 0.00018	0.00093 ± 0.000087	0.00091 ± 0.000088	0.0012 ± 0.000099	0.00088 ± 0.000047
$\gamma_{3}$	Lamb growth rate for birth rank 3, kg d^−1^	0.16 ± 0.0026	0.18 ± 0.0050	0.19 ± 0.026	0.15 ± 0.021	0.18 ± 0.039	0.26 ± 0.015	0.22 ± 0.0070	0.25 ± 0.024	0.18 ± 0.010	0.21 ± 0.0054
$\theta_{3}$	Effect of premating ewe liveweight on lamb growth rate for birth rank 3, d^−1^	0.0013 ± 0.000037	0.0011 ± 0.000073	0.0013 ± 0.00034	0.0014 ± 0.00026	0.0016 ± 0.00055	0.00058 ± 0.00018	0.00059 ± 0.000093	0.00070 ± 0.00033	0.0010 ± 0.00017	0.00066 ± 0.000085
$\gamma_{4}$	Lamb growth rate for birth rank 4, kg d^−1^	0.20 ± 0.012	0.22 ± 0.030	NA	0.75 ± 0.11	2.25 ± 0.82	NA	0.24 ± 0.020	0.068 ± 0.36	0.19 ± 0.054	0.25 ± 0.024
$\theta_{4}$	Effect of premating ewe liveweight on lamb growth rate for birth rank 4, d^−1^	0.00073 ± 0.00017	0.00023 ± 0.00043	NA	−0.0056 ± 0.0013	−0.022 ± 0.0097	NA	0.00027 ± 0.00026	0.0031 ± 0.0055	0.00076 ± 0.00090	−0.00017 ± 0.00037
$\sigma_{L}$	Perturbation amplitude for lamb growth rate, kg d^−1/2^	0.81 ± 0.0016	0.78 ± 0.0031	0.78 ± 0.0068	0.89 ± 0.0063	0.83 ± 0.0089	0.76 ± 0.0063	0.74 ± 0.0051	0.80 ± 0.0054	0.76 ± 0.0046	0.66 ± 0.0026

^*a*^ Parameters reported for mature ewes.

^*b*^ Value reported (±SEM)

^*c*^ NA indicates that there were not sufficient data to estimate the parameter.

$$\begin{array}{l} \begin{array}{ll} P(k;\bar{n},\sigma_{n}^{2},p_{n})=\\ & \frac{1}{T}\overset{7}{\underset{m=1}{\sum }}\;LN\left(m;\bar{n},\sigma_{n}^{2}\right)\frac{m!}{k!(m-k)!}\\ & \times {\left[p_{n}-d(m-1)\right]}^{k}\\ & {\left\{1-[p_{n}-d(m-1)]\right\}}^{m-k}, \end{array} \end{array}$$

$$\begin{array}{l} T=\sum {\;}_{i=1}^{7}\;LN\left(i;\bar{n},\sigma_{n}^{2}\right) \end{array}$$ (2)

where $p_{n}\;$is the probability that an embryo survives to scanning when there is initially a single ovum, *d* = 0.1 is the assumed rate of decrease in embryo survival with ovulation rate (Shorten et al., 2013), *T* is a normalization factor and the maximum ovulation rate is assumed to be seven ova. All liveweight effects occur via ovulation rate (Eq. (1)) even though liveweight is known to also affect embryo survival (Shorten et al., 2013). However, liveweight effects on ovulation are significantly greater than liveweight effects on embryo survival (Shorten et al., 2013) and therefore liveweight effects on embryo survival are not directly included in the model.

#### Conception failure to a given breeding cycle.

The model for the effect of ovulation rate (*n*) on the probability of not getting pregnant to the first cycle is

$$\begin{array}{l} P_{f}\left(n\right)=\left(1-f\right)+f{\left(1-q\right)}^{n}, \end{array}$$ (3)

where the first term denotes the probability of failure in the all-or-nothing model (Kelly and Johnstone, 1983) and the second term denotes the probability of failure in the binomial model (Restall and Griffiths, 1976) given there is no failure in the all-or-nothing model [where (1 − *q*) denotes the probability of failure for an individual ova]. It was assumed that *f* = 0.87 and *q* = 0.92 for each flock (Shorten et al., 2020).

#### Lambing day.

The day of lambing is dependent on the day of fertilization, the average gestation length (*G* = 148 d) and the standard deviation in gestation length (*σ*_g_ = 3 d). The day of lambing for a ewe that lambs to the first cycle ($d_{l}$) is

$$\begin{array}{l} d_{l}=d_{r}+u_{l}+g_{l}, \end{array}$$ (4)

$$\begin{array}{l} g_{l}∼N(G,\sigma_{g}^{2}), \end{array}$$

$$\begin{array}{l} u_{l}∼U(0,17), \end{array}$$

where $d_{r}$ is the start day of mating (assumed to be d 103 of the year), $u_{l}$ is the stage of the cycle (assumed to be uniformly distributed from 0 to 17 d), and $g_{l}$ is the gestation length (days). The day of lambing for a ewe that lambs to the second cycle is

$$\begin{array}{l} d_{l}+17, \end{array}$$ (5)

where it was assumed that ewes are exposed to the ram for two cycles.

#### Lamb survival.

Lamb survival has negative quadratic relationships with ewe premating liveweight and age (Morris et al., 2000; Everett-Hincks et al., 2014; Shorten et al., 2020). The probability of a scanned fetus surviving from scanning to weaning in a Bernoulli model of lamb survival is

$$\begin{array}{l} logit(p_{s})=\beta_{s0}+\beta_{Ws1}W+\beta_{Ws2}W^{2}+\beta_{As1}A+\beta_{As2}A^{2}, \end{array}$$ (6)

where $p_{s}$ is the probability that a lamb survives when there are initially *s* scanned fetuses.

#### Weaning weight of lambs.

Lamb growth from day born ($d_{l}$) to the day of weaning ($d_{w}$) is dependent on sex, birth rank, ewe age and weight (Thomson et al., 2004; Kenyon et al., 2004, 2009; De Blasio et al., 2007; Thompson et al., 2011; Paganoni et al., 2014; Aktas et al., 2015) and can be described by the stochastic differential equation (Shorten et al., 2020):

$$\begin{array}{l} dL=g(W,R,S)dt+\sigma_{L}dW,\;L\left(0\right)=L_{0} \end{array}$$ (7)

$$\begin{array}{l} g(W,R,S)=G_{S}+\gamma_{R}+\theta_{R}W \end{array}$$

where *L* is lamb liveweight, *t* is the time from the day of birth (days), $L_{0}$ is the birthweight, *R* is the birth rank (one to four lambs born), *W* is premating ewe liveweight, *S* is the lamb sex (M, F with $G_{F}=0$), $\gamma_{R},\theta_{R}$ are regression coefficients and *dW* is a Weiner process (i.e., a random process with independent normally distributed increments with zero mean and variance proportional to time from the day of birth) with perturbation amplitude $\sigma_{L}$. The probability distribution of weaning weight is

$$\begin{array}{ll} Y\left(L(d_{w}-d_{l})\right)= & N(L(d_{w}-d_{l});L_{0}+g(W,R,S)\\ & (d_{w}-d_{l}),(d_{w}-d_{l})\sigma_{L}^{2}/2), \end{array}$$ (8)

where $N(x;\mu ,\sigma^{2})$ denotes the normal distribution with mean μ and variance $\sigma^{2}$.

Birthweight was not measured and the mean birthweight for singles was assumed to be 5.20 kg, for twins was 4.28 kg, for triplets was 3.74 kg, for quadruplets was 3.27 kg and for quintuplets was 3.12 kg (Hinch et al., 1985; Everett-Hincks et al., 2014; Shorten et al., 2020). Ram lambs were assumed to be on average 0.25 kg heavier than ewe lambs (Oldham et al., 2011; Hocking-Edwards et al., 2019; Shorten et al., 2020). The standard deviation in lamb birthweight was assumed to be 0.80 kg for each birth rank (Shorten et al., 2020).

### Statistical Analysis

Model parameters for lamb survival were estimated with a generalized linear mixed-effects model with a binomial distribution and logit link (Lavara et al., 2011). Model factors that were not significant were not removed and other model constructions and interactions were not tested. Maximum likelihood (Pawitan, 2001) was employed to obtain estimates of ovulation rate, embryo survival, the standard deviation in ovulation rate and lamb growth rate for each flock. Calculations were conducted in Matlab (The Mathworks).

Models were parameterized for each of the nine farms and all farms combined. Separate models were constructed for mature ewes and ewe lambs. The threshold for significance is *P* < 0.05 unless stated otherwise. Root mean squared error (RMSE) was used to evaluate the accuracy of models for the distributions in a number of fetuses at scanning and premating liveweight.

The models for each reproductive step were linked to predict the flock reproduction dynamics from ovulation to weaning. Each flock was considered to have 1000 breeding ewes with distribution in age and premating liveweight representative of the flock. Day of lambing was calculated based on the assumptions that the start of mating is d 103 the year for each flock, ewes were exposed to the ram for two cycles, the standard deviation in lamb birthweight was 0.80 kg for each birth rank, and the weaning day was d 340 of the year. Simulations were repeated 10,000 times to determine the expected role of perturbations in average premating ewe liveweight, average ovulation rate, variability of ovulation rate, embryo survival, lamb survival, standard deviation in lambing day and average ewe age on the kilograms of lamb liveweight at weaning per ewe exposed to the ram (providing a total of 10 million ewes in the simulation). Simulations were conducted by considering a fixed range of deviations from the mean for each flock. Changes in the distribution of ewe age were generated with a Gamma correction ($a_{i}^{v}$) followed by normalization. Ranges (number of points over the range) were −5 to 25 kg (121) for liveweight, −0.5 to 1.0 (61) for ovulation rate, −0.5 to 0.5 (41) for standard deviation in ovulation rate, −0.2 to 0.2 (81) for embryo survival, −0.2 to 0.15 (71) for lamb survival, −0.12 to 0.13 (67) for probability of conception success, and a Gamma correction exponent (*v*) of 0.1 to 1.5 (31) for age. The perturbations in the different factors were simulated in a causal manner. For example, changes in liveweight included a future cascade of causally related changes in other reproductive variables while, for example, a change in lamb survival would not reflect changes in prior correlated, but not causal, variables such as ovulation rate.

## RESULTS AND DISCUSSION

The reproductive performance for mature ewes and ewe lambs are presented separately. Estimated model parameters are listed for each flock in Table 1 and Table S1 for mature ewes and ewe lambs, respectively.

### Mature Ewes

#### Premating liveweight.

Premating ewe liveweight was suitably described by a normal distribution. Premating ewe liveweight for each age class was also suitably described by a normal distribution. Premating ewe liveweight typically increases significantly from age 2 to 4 (Figure S1). The mean (*µ*_W,*i*_) and standard deviation (σ _W,*i*_) in weight for age class *i* for ages 2 to 9 are listed in Table 1 for each flock.

#### Ewe age distribution.

The ewe age distribution is listed in Table 1 where $a_{i}$ is the proportion of ewes in the flock of age *i* (ages 2 to 9 only).

#### Number of fetuses at mid-pregnancy.

**Figure 1.**
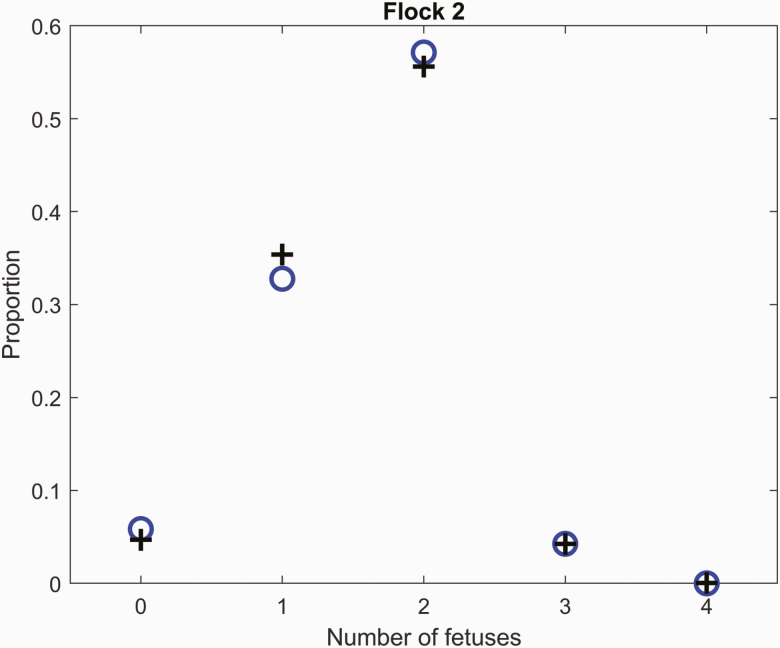
The distribution of the number of fetuses for flock 2 (o) and the compound lognormal–binomial model distribution fit (+) to the data [adjusted for age and weight effects on the number of ova via Eqs. (1) and (2)]. The mean number of fetuses was 1.60 and the standard deviation in the number of fetuses was 0.67.

The distribution of the number of fetuses at mid-pregnancy for flock 2, which is representative of the distribution in the number of fetuses scanned in a typical New Zealand flock, is shown in Figure 1. The proportions of ewes with 0, 1, 2, 3, 4, and 5 fetuses were $f_{0}$ = 0.06, $f_{1}$ = 0.33, $f_{2}$ = 0.57, $f_{3}$ = 0.04, $f_{4}$ = 0.0, and $f_{5}$ = 0.0 for flock 2. The distribution of the number of fetuses was appropriately described by a compound lognormal-binomial model adjusted for age and weight effects on the number of ova via Eqs. (1) and (2) (RMSE = 0.016 for ewes with zero to five fetuses; Figure 1).

#### Predicted ovulation rate.

**Figure 2.**
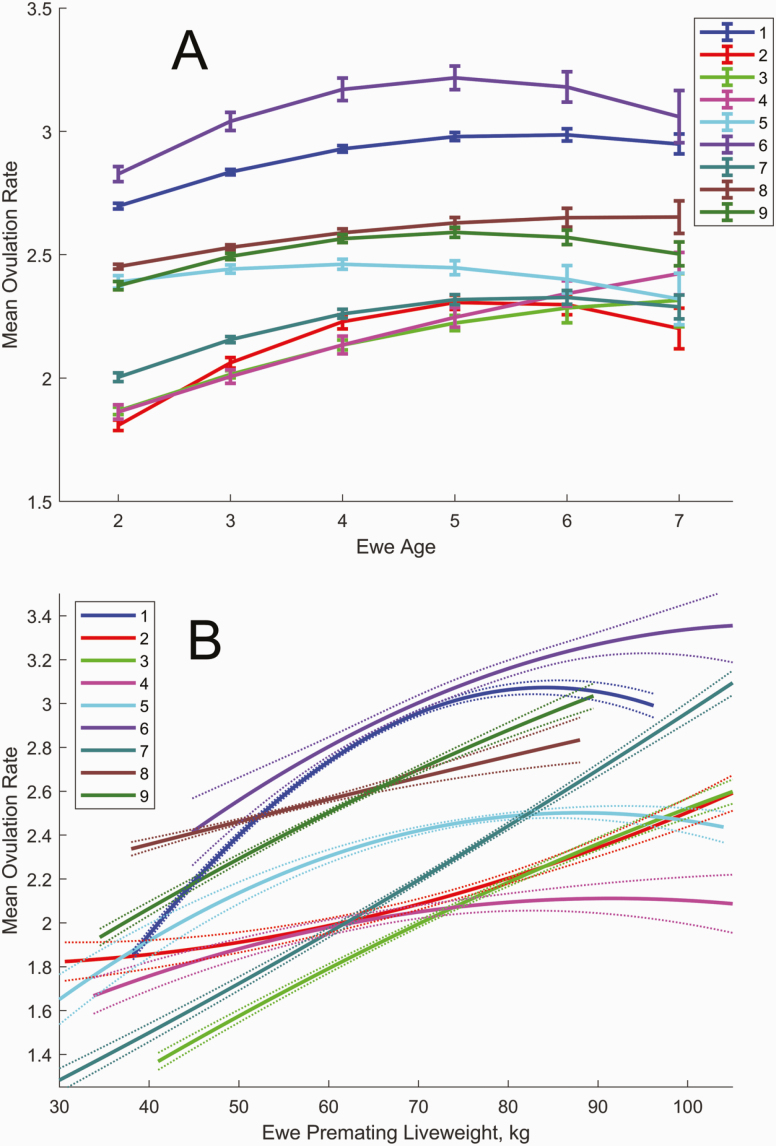
(A) Effect of ewe age on ovulation rate adjusted for premating liveweight, kg for flocks 1−9. Error bars denote SEM. (B) Effect of premating ewe liveweight on ovulation rate adjusted for ewe age for flocks 1−9. Dotted lines denote SEM. Curves are plotted over the range of values observed for each flock.

There was a significant positive effect of premating ewe liveweight (*W*) on the number of ova for all flocks combined (*P* < 0.001), consistent with estimates from other studies (Cumming 1977; Thompson et al., 1985; Davis et al., 1987). There was also a significant effect of ewe age (*A*) on the number of ova for all flocks combined (*P* < 0.001), consistent with other studies (Schoenian and Burfening 1990; Shorten et al., 2020). The effects of age and premating ewe liveweight on ovulation rate was variable between flocks although ovulation rate typically increased with liveweight, and peak ovulation rate occurred at age 5 in most flocks (Figure 2). Mean ovulation rate varied from two to three ova between flocks (consistent with New Zealand research flocks (Davis et al., 1987; Shorten et al., 2013)) and this was associated with mean scanning rates of 1.6 to 2.4 fetuses between flocks. The estimated probability of embryo survival to scanning varied from 0.88 to 0.99 between flocks (Table 1) for ewes with a single embryo (consistent with direct estimates of embryo survival probability for ewes from research flocks (Shorten et al., 2013)), although these indirect estimates are conditional on the assumed lognormal distribution for ovulation rate, the binomial embryo survival process and the assumed 0.1 decrease in embryo survival probability with ovulation rate (embryo survival estimates will be biased for flocks that significantly deviate from these model assumptions).

#### Lambing day.

Day of lambing is dependent on the average gestation length (*G* = 148 d), standard deviation in gestation length (*σ*_g_ = 3 d) and the day of fertilization. The average day of lambing was d 262 of the year (for all ewes of all age classes) and varied from d 247 to 275 between flocks. The mean and variance in the day of lambing will also be dependent on the number of cycles that ewes were exposed to the ram and variation between years in the date ewes were exposed to the ram, which were not recorded. Ewes were assumed to be exposed to the ram for two cycles.

**Figure 3.**
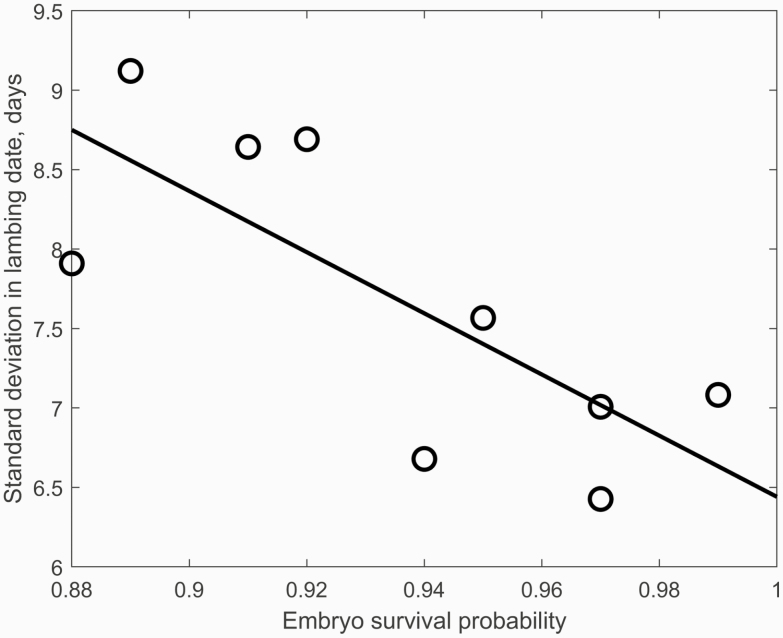
The relationship between estimated embryo survival probability from ovulation to scanning for ewes with a single ovum and the standard deviation in lambing day for each flock (*R*^2^ = 0.58; *P* = 0.02; RMSE = 0.67 d).

However, between year variability in lambing dates were relatively low within each flock (*σ*_year_ = 3 d). A standard deviation in lambing day of 4.9 d is expected if all ewes lamb to the first cycle they were exposed to the ram (assuming *G* = 148 d for all ewes). The relationship between estimated embryo survival probability from ovulation to scanning and the standard deviation in lambing day for each flock is shown in Figure 3. There was a decrease in the standard deviation in the lambing day of 0.2 d per % increase in embryo survival probability. Note that the estimate of embryo survival probability was obtained with data that included ewes that had zero fetuses. Embryo loss is a nonlinear function of time from ovulation, with greater losses in early pregnancy. Embryo survival is therefore expected to be a predictor of the proportion of ewes that get pregnant to the second cycle exposed to the ram (and lamb 17 d later on average than ewes that get pregnant to the first cycle exposed to the ram). The minimum achievable standard deviation in lambing day is 5.75 d assuming *σ*_year_ = 0 d and is 6.5 d assuming σ _year_ = 3 d (both assuming *σ*_g_ = 3 d and a 17-d cycle), which is consistent with minimum values in Figure 3. These results are consistent with tighter lambing day distribution in flocks with high embryo survival.

#### Lamb survival.

**Figure 4.**
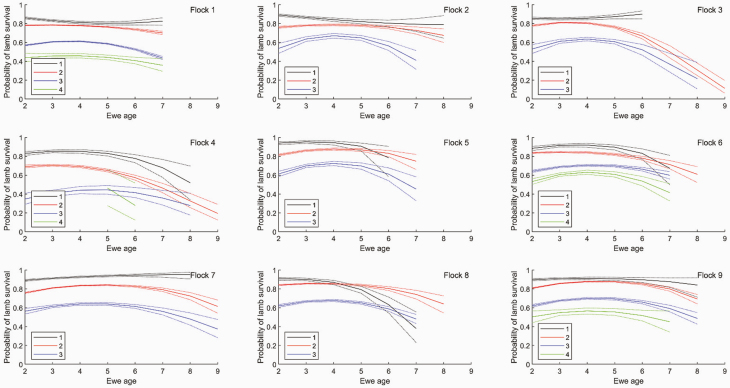
The effect of ewe age on the probability of lamb survival for 1−4 fetuses adjusted for premating liveweight for each flock. Dotted lines denote SEM. Curves are plotted over the range of values observed for each flock.

The relationship between the number of fetuses scanned and the number of lambs weaned was consistent with a Binomial model for lamb survival. Lamb survival probability for triplets was significantly lower than that for twins, which was significantly lower than that for singles (*P* < 0.001), consistent with other studies (Morris et al., 2000; Everett-Hincks et al., 2014). Lamb survival was typically 88%, 81%, 62%, 53% for ewes with 1, 2, 3, 4 fetuses, respectively, although lamb survival varied by up to 10% between flocks (Figure 4). Lamb survival was not significantly different between triplets and quadruplets. There was a significant quadratic effect of premating ewe liveweight on lamb survival consistent with the increased metabolic demand on ewes with multiple lambs (Kenyon et al., 2013, 2019), with lower survival for low and high premating ewe liveweight, although the strength of this effect varied between flocks. Ewes that weaned more lambs than were recorded mid-pregnancy were omitted from the analysis (2% of records).

The effect of ewe age on lamb survival as a function of the number of fetuses is shown in Figure 4 for each flock. There was a significant effect of animal age on the probability of lamb survival. Lamb survival for twins was greatest for 4-yr-old ewes and decreased in older ewes, consistent with Morris et al., (2000) and Everett-Hincks et al., (2014). Lamb survival for twins was not significantly lower in 2-yr-old than 3- or 4-yr-old ewes. Lamb survival for twins decreased significantly from age 4 (*P* < 0.01).

**Figure 5.**
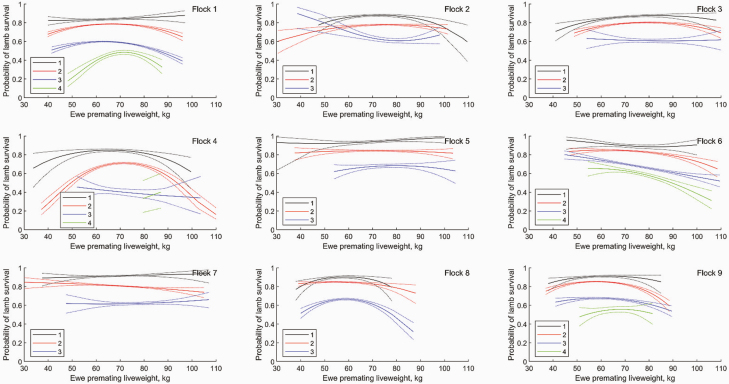
The effect of ewe premating liveweight on the probability of lamb survival for 1−4 fetuses (adjusted for ewe age effects for each flock). Dotted lines denote SEM. Curves are plotted over the range of values observed for each flock.

The effect of premating liveweight on the probability of lamb survival is also shown in Figure 5 for different scanning rates. The optimal ewe liveweight for lamb survival tended to increase with litter size (although this was flock dependent), where the optimal premating liveweight was 65, 72 kg for ewes with one, two lambs, respectively, in flock 4. The optimal premating liveweight for ewes with twins varied between flocks from 50 to 80 kg.

#### Weaning weight of lambs.

**Figure 6.**
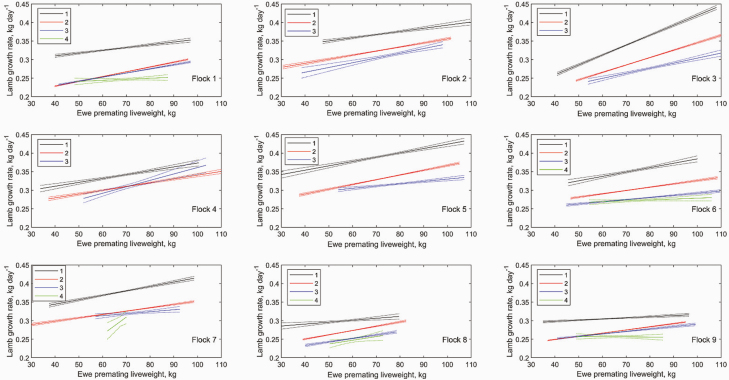
The effect of premating ewe liveweight on lamb growth rate for 1−4 fetuses for all ewes in each flock (1−9). Dotted lines denote SEM. Curves are plotted over the range of values observed for each flock.

Lamb growth rate is significantly greater for lambs of lower birth rank (*P* < 0.001) (Figure S2). There were significant effects of premating ewe liveweight on the lamb growth rate for singles, twins, triplets and quadruplets respectively (*P* < 0.001). This effect was greatest for the lambs of lower birth rank (*P* < 0.001). There was significant between flock variability in both lamb growth rate and the effect of premating ewe liveweight on lamb growth rate (Figure 6).

Single lambs had a 54 g d^−1^ growth advantage over twin lambs, consistent with other studies (Kenyon et al., 2004; Thompson et al., 2011; Pagonini et al., 2014; Shorten et al., 2020). Twin lambs had a 22 g d^−1^ growth advantage over triplet lambs, which is lower than other studies (Muir et al., 2003; Pagonini et al., 2014), although this difference is dependent on premating ewe liveweight and is variable between flocks (Kenyon et al., 2019). Triplet lambs tended to have an 11 g d^−1^ growth advantage over quadruplet lambs.

#### Body condition score.

The changes in BCS from mating to scanning for flock 6 were 0.11 ± 0.02, 0.09 ± 0.006, 0.03 ± 0.006, −0.11 ± 0.01, −0.23 ± 0.06 for all ewe age classes (excluding ewe lambs) that scanned one to five fetuses, respectively (only able to be estimated for flock 6). Ewes with four or five fetuses tended to lose BCS from mating to scanning. These changes in BCS from mating to scanning are therefore less than the 0.25 BCS decrease from premating to scanning in mature ewes observed by Pettigrew et al., (2020) irrespective of a number of lambs born (1 to 5).

The decrease in BCS from scanning to weaning were 0.04 ± 0.02, 0.42 ± 0.007, 0.49 ± 0.01, 0.49 ± 0.02, 0.47 ± 0.08 in ewes with one, two, three, four, five fetuses scanned, respectively (only able to be estimated for flock 6 for all ewes age classes excluding ewe lambs). There was therefore a very small decrease in BCS from scanning to weaning in ewes with a single fetus scanned and a 0.42 to 0.47 BCS decrease from scanning to weaning in ewes with multiple fetuses. This is similar to the 0.42 BCS decrease in mature ewes from premating to weaning observed by Pettigrew et al., (2020).

An increase in one BCS at mating equates on average to 8.45 ± 0.07 kg liveweight at mating (all flocks; *n* = 53363, all ewes excluding ewe lambs) and 7.21 ± 0.07, 8.83 ± 0.16, 4.99 ± 0.15, and 5.33 ± 0.13 kg liveweight at mating in flocks 1, 6, 8, 9, respectively, (RMSE = 5.9, 8.0, 6.4, and 6.1 kg, respectively) and was not able to be estimated for other flocks. These estimates are consistent with the difference of 7.3 kg per unit BCS observed by Kenyon et al., (2004).

For each unit decrease in BCS from mating to weaning there was a small associated decrease in BCS (0.15 ± 0.01 BCS) and liveweight (1.3 ± 0.2 kg) at mating the following year (flock 6; ewes of all age classes excluding ewe lambs). This indicated that ewes that lost body condition in a given year (due to rearing multiple lambs) largely, but not completely, recovered lost body condition by mating the next year under the farm system for flock 6 (i.e., there was enough pasture, time and genetic potential to recover BCS by mating in the following year).

### Ewe Lamb Reproductive Performance

#### Premating liveweight.

Premating ewe lamb liveweight was be suitably described by a normal distribution (Supplementary Figure S3). The mean (µ _W,i_) and standard deviation (*σ*_W,*i*_) in liveweight for ewe lambs (age class *i* = 1) are listed in Supplementary Table S1 for each flock.

#### Ewe age distribution.

The ewe age distribution is listed in Supplementary Table S1, where $a_{i}$ is the proportion of ewes in the flock of age *i* (ages 1 to 9 only). Ewes in Supplementary Table S1 include animals that were exposed to the ram (some ewe lambs were not exposed to the ram and therefore were not considered for analysis of reproductive traits).

#### Number of fetuses at mid-pregnancy.

The distribution of the number of fetuses for flock 2 is shown in Supplementary Figure S4. The proportions of ewe lambs with 0, 1, 2, 3, and 4 fetuses were $f_{0}$ = 0.43, $f_{1}$ = 0.42, $f_{2}$ = 0.15, $f_{3}$ = 0. 0009, and $f_{4}$ = 0.0 for flock 2 (RMSE = 0.04 for ewes with zero to four fetuses; Supplementary Figure S4).

#### Predicted ovulation rate.

**Figure 7.**
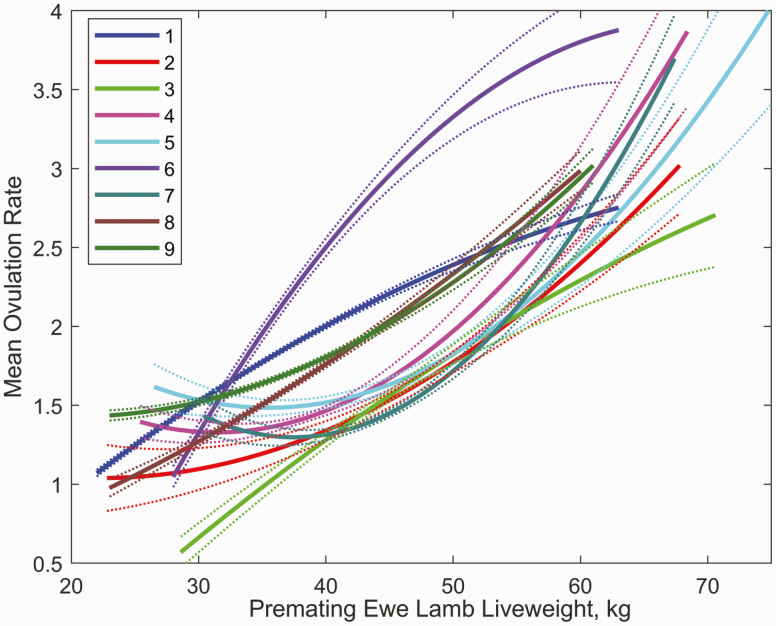
Effect of premating ewe lamb liveweight on the ovulation rate for each flock. Dotted lines denote SEM. Curves are plotted over the range of values observed for each flock.

**Figure 8.**
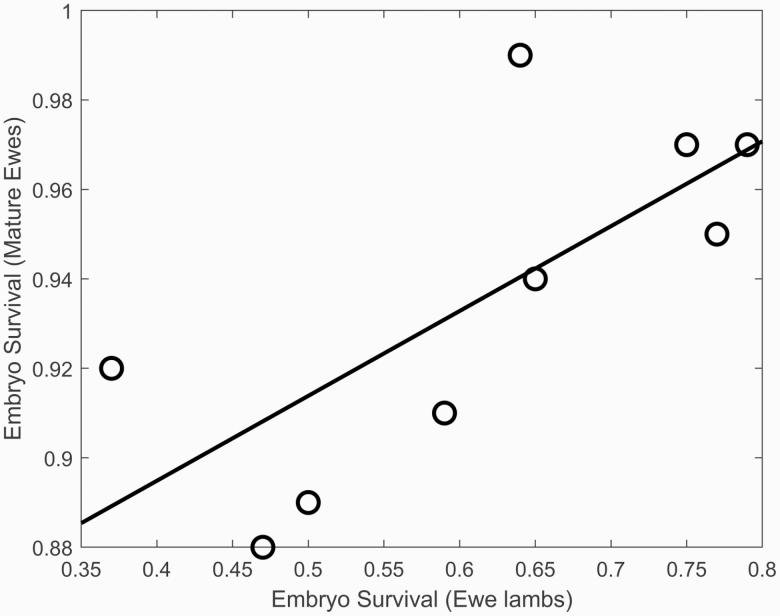
The relationship between embryo survival from ovulation to scanning in ewe lambs and mature ewes with a single ovum for each flock (*R*^2^ = 0.53; *P* = 0.03; RMSE = 0.03; slope = 0.19 ± 0.07; intercept = 0.82 ± 0.04).

There was a significant positive effect of premating ewe lamb liveweight on the number of ova for all flocks combined (*P* < 0.0001). The effect of premating ewe lamb liveweight on ovulation rate was highly variable between flocks although ovulation rate typically increased with liveweight (Figure 7). Liveweight had a significant effect on the number of fetuses mid-pregnancy with a 2−3-fold increase for ewe lambs with premating liveweights of 60 kg compared to 30 kg. Mean ovulation rate varied from 1.5 to 2.25 ova between flocks and was associated with mean scanning rates of 0.46 to 1.33 fetuses between flocks. Flocks with high ewe lamb ovulation rate tended to be the flocks with high mature ewe ovulation rate as expected (flocks 6, 1, 8, 9). The probability of embryo survival to scanning varied from 0.37 to 0.77 between flocks for ewe lambs with a single embryo (Figure 8), with similar between flock differences in embryo survival for greater ovulation rates, although these estimates were conditional on the assumed lognormal distribution for ovulation rate and binomial embryo survival process. Note that the embryo survival and ovulation rate estimates were also conditional on the assumption that all ewe lambs are ovulating at mating. Not all ewe lambs achieve puberty at mating and it has been estimated that 82% of ewe lambs achieve puberty in their first year of life in a New Zealand research flock, although this is variable between years (Edwards et al., 2015) and is likely to be variable between farms. This is also consistent with the 89% of Merino ewe lambs that attained puberty at 250 d of age in a single year (Nieto et al., 2013). If it is assumed that 82% of ewe lambs achieve puberty at mating, then the estimated embryo survival probability increased from 0.63 to 0.82 (all flocks). If the proportion of ewe lambs that achieve puberty at mating was included as an extra parameter in the estimation procedure then the maximum likelihood estimated embryo survival probability was 0.73 and the percentage of ewe lambs achieving puberty at mating was 87% (all flocks), consistent with research flock estimates for the percentage of ewe lambs achieving puberty at mating (Nieto et al., 2013; Edwards et al., 2015).

The relationship between embryo survival from ovulation to scanning in ewe lambs and mature ewes with a single ovum is shown in Figure 8 for each flock. Flocks with high embryo survival ewe lambs tended to have high embryo survival mature ewes as expected. The between flock variability in embryo survival was five-fold greater for ewe lambs than mature ewes.

#### Lamb survival.

Lamb survival probability for triplets was significantly lower than that for twins, which was significantly lower than that for singles (*P* < 0.001). Lamb survival was typically 81%, 71%, 43%, 38% for ewe lambs with one, two, three, four fetuses, respectively, although lamb survival varied by up to 10% between flocks (Supplementary Figure S5). There was no clear relationship between lamb survival for ewe lambs and lamb survival for mature ewes, although lamb survival in ewe lambs was more variable between flocks than for mature ewes. Lamb survival was not significantly different between triplets and quadruplets. There was a significant quadratic effect of premating ewe lamb weight on lamb survival, although the strength of this effect varied between flocks. Ewe lambs that weaned more lambs than fetuses scanned were omitted from the analysis (2% of records).

The effect of premating liveweight on the probability of lamb survival for six flocks are also shown in Supplementary Figure S5 for different scanning rates. Lamb survival for singles was greatest for ewe lambs with lower liveweights in flock 6, although the relationship between liveweight and lamb survival varied between flocks (some of this variability is due to low sample sizes).

#### Weaning weight of lambs.

Lamb growth rate was significantly greater for lambs of lower birth rank (*P* < 0.001) (Supplementary Figure S6). There were significant effects of premating ewe lamb liveweight on lamb growth rate for singles and twins, respectively (*P* < 0.001). There was significant between flock variability in both the lamb growth rate and the effect of premating ewe lamb liveweight on lamb growth rate (Supplementary Figure S6).

#### Body condition score.

BCS changes from mating to scanning were not able to be estimated for ewe lambs. BCS changed 0.02 ± 0.03, −0.30 ± 0.02, −0.39 ± 0.04, and 0.15 ± 0.15 BCS from scanning to weaning in ewe lambs with one, two, three, four fetuses, respectively (only able to be estimated for flock 6 for ewe lambs). BCS was unchanged from scanning to weaning in ewe lambs with a single lamb born and decreased 0.3 BCS from scanning to weaning in ewe lambs with multiple lambs born. This is similar to the 0.24 BCS decrease in ewe lambs from premating to weaning observed by Pettigrew et al., (2020).

An increase in 1 BCS at mating equated on average to 4.58 ± 1.20 kg liveweight at mating (flock 6; *n* = 106, RMSE = 3.4 kg, all ewe lambs) and was not able to be estimated for other flocks.

For each unit decrease in BCS from mating to weaning there was a small associated decrease in BCS (0.31 ± 0.12 BCS) at mating the following year (flock 6; ewe lambs). This indicated that ewe lambs that lost body condition in a given year (due to rearing multiple lambs) largely, but not completely, recovered lost body condition by mating the next year under the farm system for flock 6 (i.e., there was enough pasture, time and genetic potential to recover BCS by mating in the following year).

#### Simulation of the flock reproduction dynamics—mature ewes.

**Figure 9.**
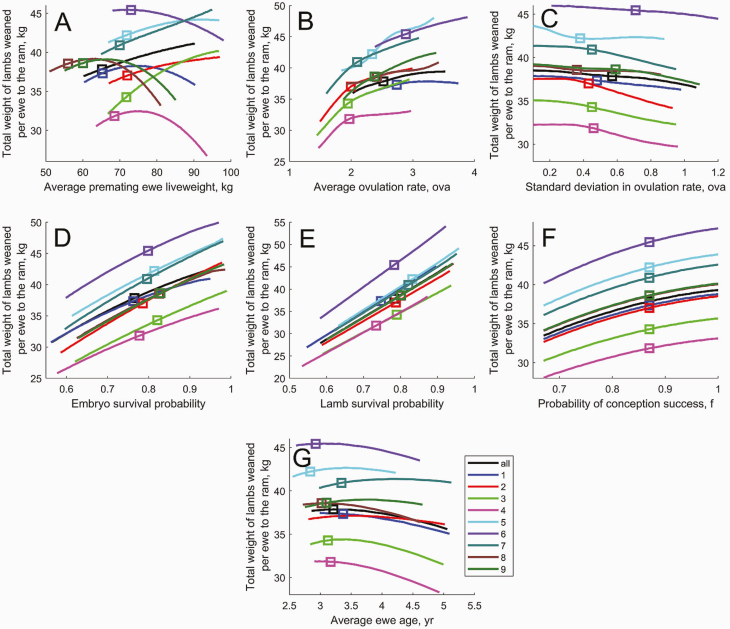
The effect of change in the (A) average premating ewe liveweight, (B) average ovulation rate, (C) standard deviation in ovulation rate, (D) embryo/fetal survival probability, (E) lamb survival probability, (F) conception success (*f*), and (G) average ewe age on the total weight of lambs weaned per ewe exposed to the ram (for mature ewes in flocks 1−9). Square symbols denote the current flock average.

We investigated the effect of average premating ewe liveweight, average ovulation rate, variability of ovulation rate, embryo survival, lamb survival, standard deviation in lambing day and average ewe age on the kilograms of lamb liveweight at weaning per ewe exposed to the ram (Figure 9 for each flock). The effects of perturbations in different factors on the kilograms of lamb liveweight at weaning per ewe exposed to the ram were investigated using the elasticity metric, which is a unitless ratio of the percentage change in one variable to the percentage change in a second variable, when the second variable has a causal influence on the former. The elasticities describe the relative importance of the effect of average premating ewe liveweight (0.30), average ovulation rate (0.19), variability of ovulation rate (−0.024), embryo survival (0.60), lamb survival (1.03), conception failure (0.36) and average ewe age (0.02) on the kilograms of lamb liveweight at weaning per ewe exposed to the ram (based on all flocks combined). The largest elasticity was for lamb survival and indicated that a 1% increase in lamb survival was associated with a 1.03% increase in the total kilograms of lamb liveweight at weaning per ewe exposed to the ram. Furthermore, the elasticity for embryo survival was 60% of that for lamb survival for these flocks. The elasticities for each flock are listed in Table 2 and the flock statistics for these simulations are listed in Table 3 (the product of the average number of fetuses scanned, average lamb survival and average weaning weight provides an estimate of the average total weight of lambs weaned per ewe exposed to the ram, although this approximation ignores, for example, covariances between these different random variables). For example, the elasticity for premating ewe liveweight was −0.03 for flock 6, which is near optimal, whereas the elasticity was 0.84 for flock 3, indicating that a 10 kg increase in premating ewe liveweight will generate an 8 kg increase in the total weight of lambs weaned per ewe exposed to the ram. This indicated that premating liveweight ranged from optimal to suboptimal between flocks and that for these suboptimal farms there is potential to increase flock performance through improved management of ewe premating liveweight. The elasticities for ovulation rate were highly variable between flocks (0.16 to 0.50 for mature ewes), indicating that ovulation rate was near-optimal for some flocks, whereas there was potential to improve flock performance in suboptimal flocks. These elasticities can be used to assess the cost–benefit of different factors on the reproductive performance of each flock, which is dependent on the cost of the intervention and is variable between factors. However, assuming similar costs of intervention for each factor, this indicates that increasing lamb survival for each flock will provide the largest increase in the kilograms of lamb liveweight at weaning per ewe exposed to the ram, which is consistent with other economic evaluation studies (Amer et al., 1999; Young et al., 2011, 2014).

**Table 2.** Table of elasticities for mature ewes

Parameter	All flocks	Flock 1	Flock 2	Flock 3	Flock 4	Flock 5	Flock 6	Flock 7	Flock 8	Flock 9
Premating ewe liveweight	0.30	0.32	0.34	0.84	0.41	0.30	−0.03	0.36	0.23	0.22
Mean ovulation rate	0.19	0.16	0.38	0.42	0.30	0.50	0.22	0.34	0.19	0.33
Standard deviation in ovulation rate	−0.025	−0.025	−0.047	−0.048	−0.051	−0.022	−0.021	−0.033	−0.011	−0.026
Embryo survival	0.60	0.54	0.77	0.73	0.65	0.63	0.56	0.70	0.66	0.70
Lamb survival	1.03	1.04	1.02	1.02	1.05	1.03	1.03	1.03	1.03	1.03
Probability of conception success	0.36	0.37	0.37	0.36	0.37	0.37	0.36	0.36	0.36	0.36
Ewe age	0.01	−0.04	0.05	0.07	−0.04	0.09	0.00	0.10	0.00	0.09

**Table 3.** Table of flock statistics for mature ewes

Parameter	All flocks	Flock 1	Flock 2	Flock 3	Flock 4	Flock 5	Flock 6	Flock 7	Flock 8	Flock 9
Premating ewe liveweight, kg	65	65	72	72	69	72	73	70	56	60
Mean ovulation rate, ova	2.53	2.74	1.99	1.94	1.97	2.34	2.89	2.10	2.42	2.38
Standard deviation in ovulation rate, ova	0.57	0.48	0.43	0.45	0.46	0.38	0.71	0.45	0.36	0.59
Embryo survival probability	0.77	0.76	0.79	0.82	0.78	0.81	0.80	0.80	0.83	0.83
Lamb survival probability	0.78	0.75	0.79	0.79	0.73	0.83	0.78	0.82	0.80	0.80
Probability of conception success	0.87	0.87	0.87	0.87	0.87	0.87	0.87	0.87	0.87	0.87
Ewe age, yr	3.2	3.4	3.1	3.1	3.2	2.8	2.9	3.3	3.0	3.1
Average lamb growth rate, kg d^−1^	0.28	0.27	0.34	0.30	0.32	0.30	0.29	0.34	0.27	0.27
Average weaning weight, kg	26.6	25.4	30.7	28.0	29.8	27.9	26.9	31.0	25.2	25.6
Average number of fetuses scanned	1.89	2.06	1.57	1.59	1.54	1.88	2.25	1.66	1.97	1.94
Average total weight of lambs weaned per ewe exposed to the ram, kg	37.8	37.3	37.0	34.2	31.8	42.2	45.4	40.9	38.5	38.6

The calculated elasticities provide information on the expected change in flock performance and are therefore suitable for the strategic management of the flock. Although between year variation was not explicitly examined in this study, the between year variation in birthweight, litter size and lamb survival has an estimated coefficient of variation of 10% to 15 % (Kleeman et al. 1990). Within year tactical decision making is required by the farmer to optimize reproductive performance due to variation in environmental and other factors that occur with a year. This complex decision-making process, which is subject to uncertain and partial information, was not considered. The quantity and quality of data collection are also highly variable between commercial farms and is an important consideration for the application of this model to individual farms.

#### Simulation of the flock reproduction dynamics—ewe lambs.

We investigated the effect of average premating ewe lamb liveweight, average ovulation rate, variability of ovulation rate, embryo survival, lamb survival, and standard deviation in lambing day on the kilograms of lamb liveweight at weaning per ewe lamb exposed to the ram (Supplementary Figure S7 for flocks 1−4, 6, 9). The effects of perturbations in different factors on the kilograms of lamb liveweight at weaning per ewe lamb exposed to the ram were investigated using the elasticity metric. The elasticities describe the relative importance of the effect of average premating ewe lamb liveweight (−0.18), average ovulation rate (0.28), variability of ovulation rate (−0.021), embryo survival (0.83), lamb survival (1.02), and conception failure (0.35) on the kilograms of lamb liveweight at weaning per ewe exposed to the ram (all flocks). However, flock 1 contained a much larger number of ewe lamb records compared to other flocks (see estimated standard errors in Supplementary Table S1) and for this reason, the averages and elasticities for all flocks were heavily weighted towards flock 1. The elasticities for each individual flock are listed in Table 4 and the flock statistics for these simulations are listed in Table 5. The largest elasticity was typically for lamb survival, although for 50% of flocks the largest elasticity was for premating ewe lamb liveweight. This highlights the key importance of ewe lamb premating liveweight in some flocks and that ewe lamb mating is better suited to some flocks. The elasticities for ewe lamb premating liveweight varied from −0.18 to 1.39, indicating that premating liveweight ranged from optimal to suboptimal between flocks. For these suboptimal farms, the opportunity exists to increase flock performance through improved management of ewe lamb premating liveweight. Flocks 1 and 6 tended to have the lowest elasticities for premating liveweight, ovulation rate and embryo survival for ewe lambs, and was consistent with the corresponding mature ewe elasticities for flocks 1 and 6 as expected.

**Table 4.** Table of elasticities for ewe lambs^*a*^

Parameter	All flocks	Flock 1	Flock 2	Flock 3	Flock 4	Flock 6	Flock 9
Premating ewe lamb liveweight	−0.18	−0.18	1.39	1.27	1.48	0.53	0.43
Mean ovulation rate	0.28	0.40	0.47	0.47	0.52	0.31	0.49
Standard deviation in ovulation rate	−0.021	−0.046	−0.076	−0.063	−0.032	−0.058	−0.11
Embryo survival	0.83	0.84	0.92	0.99	0.90	0.81	0.77
Lamb survival	1.02	1.02	1.03	1.01	1.02	1.02	1.02
Probability of conception success	0.35	0.35	0.36	0.37	0.38	0.36	0.36

^*a*^ There were not sufficient data to calculate the elasticity for flocks 5, 7, and 8.

**Table 5.** Table of flock statistics for ewe lambs^*a*^

Parameter	All flocks	Flock 1	Flock 2	Flock 3	Flock 4	Flock 5	Flock 6	Flock 7	Flock 8	Flock 9
Premating ewe lamb liveweight, kg	42	41	50	46	43	47	41	48	41	41
Mean ovulation rate, ova	2.29	2.01	1.76	1.52	1.51	1.39	2.40	1.78	1.82	1.82
Standard deviation in ovulation rate, ova	0.47	0.42	0.48	0.36	0.28	0.34	0.63	0.32	0.27	0.45
Embryo survival probability	0.50	0.55	0.42	0.31	0.41	0.73	0.50	0.51	0.67	0.70
Lamb survival probability	0.73	0.77	0.73	0.83	0.71	NA	0.88	NA	NA	0.68
Probability of conception success	0.87	0.87	0.87	0.87	0.87	0.87	0.87	0.87	0.87	0.87
Average lamb growth rate, kg d^−1^	0.23	0.22	0.25	0.29	0.26	NA	0.24	NA	NA	0.25
Average weaning weight, kg	22.6	21.7	24.6	27.8	25.3	NA	23.6	NA	NA	23.9
Average number of fetuses scanned	1.10	1.08	0.71	0.46	0.62	0.98	1.14	0.87	1.18	1.31
Average total weight of lambs weaned per ewe exposed to the ram, kg	17.7	17.5	12.4	10.5	10.8	NA	22.7	NA	NA	20.8

^*a*^ NA indicates that there were not sufficient data to calculate the statistic.

#### Comparison of reproduction loss between mature ewes and ewe lambs.

Reproductive loss was significantly greater in ewe lambs than mature ewes, although the relative difference was dependent on the stage of reproduction and was variable between flocks. Scanning rate was 55% lower for ewe lambs. Predicted ovulation rate was 25% lower for ewe lambs. There was a 30% relative decrease in the predicted embryo survival probability from ovulation to scanning for ewe lambs. There was a 10% relative decrease in lamb survival probability from birth to weaning for ewe lambs. Lamb growth was also 25% lower for ewe lambs. This is consistent with the 45% to 50% reduction in lambs to weaning (Kenyon et al., 2004; Edwards et al., 2016), the 15% to 33% reduction in ovulation rate (Beck et al., 1996; Edwards et al., 2016), the 22% to 26% relative reduction in embryo survival probability from ovulation to scanning (Beck et al., 1996; Edwards et al., 2016), the 3% relative reduction in lamb survival probability from scanning to weaning (Edwards et al., 2016; Pettigrew et al., 2020), and the 16% reduction in lamb growth rate (Pettigrew et al., 2020) for ewe lambs compared to mature ewes in other flocks (Edwards et al., 2016).

## Supplementary Material

txab013_suppl_Supplementary_Materials
