# Supplementary material for: The role of reproductive loss on flock performance: a comparison of nine industry flocks
Source: Transl Anim Sci. 2021 Jan 28;5(1):txab013. doi: 10.1093/tas/txab013 (PMC7963042; doi:10.1093/tas/txab013)

**Supplemental Data:**

Supplementary data are available online.

Table S1: Table of model parameters, values and descriptions for ewe lambs.

| Parameter | Description | All Flocks ^1,2,3^ | Flock 1 | Flock 2 | Flock 3 | Flock 4 | Flock 5 | Flock 6 | Flock 7 | Flock 8 | Flock 9 |
| --- | --- | --- | --- | --- | --- | --- | --- | --- | --- | --- | --- |
| $\beta_{n0}$ | Intercept term for the relationship between age and weight on ovulation rate, ova | -2.70 ± 0.019 | -0.48 ± 0.051 | 1.51 ± 0.63 | -1.65 ± 0.30 | 3.16 ± 0.42 | 3.54 ± 0.98 | -4.31 ± 0.18 | 5.06 ± 0.71 | 0.29 ± 0.25 | 1.81 ± 0.082 |
| $\beta_{w1}$ | Linear effect of pre-mating liveweight on ovulation rate, ova kg^-1^ | 0.21 ± 0.0017 | 0.081 ± 0.0033 | -0.043 ± 0.027 | 0.088 ± 0.015 | -0.12 ± 0.021 | -0.12 ± 0.047 | 0.24 ± 0.012 | -0.20 ± 0.032 | 0.020 ± 0.013 | -0.038 ± 0.0051 |
| $\beta_{w2}$ | Quadratic effect of pre-mating liveweight on ovulation rate, ova kg^-2^ | -0.0021 ± 0.000034 | -0.00047 ± 0.000061 | 0.00096 ± 0.00032 | -0.00038 ± 0.00022 | 0.0019 ± 0.00031 | 0.0016 ± 0.00056 | -0.0018 ± 0.00022 | 0.0027 ± 0.00036 | 0.00041 ± 0.00018 | 0.00095 ± 0.000091 |
| $\sigma_{n}$ | Standard deviation in ovulation rate, ova | 0.47 ± 0.0088 | 0.42 ± 0.013 | 0.48 ± 0.064 | 0.36 ± 0.038 | 0.28 ± 0.056 | 0.34 ± 0.035 | 0.63 ± 0.048 | 0.32 ± 0.036 | 0.37 ± 0.017 | 0.45 ± 0.011 |
| $p_{n}$ | Probability of embryo survival to scanning for single ovulation | 0.63 ± 0.0020 | 0.65 ± 0.0036 | 0.50 ± 0.0091 | 0.37 ± 0.0076 | 0.47 ± 0.0093 | 0.77 ± 0.02 | 0.64 ± 0.0065 | 0.59 ± 0.011 | 0.75 ± 0.0083 | 0.79 ± 0.0059 |
|  |  |  |  |  |  |  |  |  |  |  |  |
| µ_W,1_ | Mean pre-mating liveweight for 1 year old ewe, kg | 42.3 ± 0.03 | 40.8 ± 0.05 | 50.4 ± 0.13 | 45.9 ± 0.11 | 43.3 ± 0.13 | 46.9 ± 0.21 | 40.8 ± 0.07 | 48.0 ± 0.14 | 40.6 ± 0.10 | 41.3 ± 0.05 |
| σ_W,1_ | Standard deviation in pre-mating liveweight for 1 year old ewe, kg | 6.01 ± 0.02 | 5.85 ± 0.03 | 6.19 ± 0.09 | 5.34 ± 0.08 | 6.61 ± 0.09 | 5.07 ± 0.21 | 4.52 ± 0.05 | 5.47 ± 0.10 | 5.04 ± 0.07 | 4.00 ± 0.03 |
|  |  |  |  |  |  |  |  |  |  |  |  |
| $a_{1}$ | Proportion of ewes in the flock of age 1 | 0.194 | 0.154 | 0.206 | 0.186 | 0.211 | 0.358 | 0.212 | 0.073 | 0.351 | 0.414 |
| $a_{2}$ | Proportion of ewes in the flock of age 2 | 0.316 | 0.302 | 0.321 | 0.321 | 0.281 | 0.316 | 0.372 | 0.335 | 0.281 | 0.251 |
| $a_{3}$ | Proportion of ewes in the flock of age 3 | 0.217 | 0.223 | 0.222 | 0.227 | 0.245 | 0.182 | 0.216 | 0.241 | 0.180 | 0.155 |
| $a_{4}$ | Proportion of ewes in the flock of age 4 | 0.140 | 0.151 | 0.131 | 0.152 | 0.158 | 0.090 | 0.119 | 0.163 | 0.109 | 0.095 |
| $a_{5}$ | Proportion of ewes in the flock of age 5 | 0.078 | 0.088 | 0.069 | 0.084 | 0.066 | 0.038 | 0.052 | 0.106 | 0.054 | 0.052 |
| $a_{6}$ | Proportion of ewes in the flock of age 6 | 0.038 | 0.050 | 0.040 | 0.022 | 0.031 | 0.013 | 0.022 | 0.055 | 0.020 | 0.025 |
| $a_{7}$ | Proportion of ewes in the flock of age 7 | 0.014 | 0.023 | 0.0080 | 0.0058 | 0.0072 | 0.0018 | 0.0058 | 0.020 | 0.0043 | 0.0080 |
| $a_{8}$ | Proportion of ewes in the flock of age 8 | 0.0038 | 0.0081 | 0.0016 | 0.0024 | 0.00047 | 0 | 0.00049 | 0.0054 | 0.00036 | 0.00052 |
| $a_{9}$ | Proportion of ewes in the flock of age 9 | 0.00034 | 0.00031 | 0.00030 | 0.00017 | 0.000052 | 0 | 0 | 0.0016 | 0 | 0 |
|  |  |  |  |  |  |  |  |  |  |  |  |
| $\beta_{10}$ | Intercept term for the relationship between age and weight and lamb survival for singles | 12.3 ± 1.16 | 22.2 ± 2.16 | 1.60 ± 5.56 | 6.88 ± 7.12 | -12.7 ± 4.76 | NA | 1.95 ± 7.61 | NA | NA | 10.7 ± 7.24 |
| $\beta_{W11}$ | Linear effect of pre-mating liveweight on lamb survival for singles, kg^-1^ | -0.46 ± 0.052 | -0.91 ± 0.10 | -0.027 ± 0.22 | -0.20 ± 0.29 | 0.58 ± 0.20 | NA | 0.11 ± 0.36 | NA | NA | -0.48 ± 0.36 |
| $\beta_{W12}$ | Quadratic effect of pre-mating liveweight on lamb survival for singles, kg^-2^ | 0.0046 ± 0.00058 | 0.0097 ± 0.0012 | 0.00036 ± 0.0021 | 0.0019 ± 0.0030 | -0.0059 ± 0.0021 | NA | -0.0023 ± 0.0041 | NA | NA | 0.0058 ± 0.0045 |
| $\beta_{20}$ | Intercept term for the relationship between age and weight and lamb survival for twins | 10.4 ± 0.89 | 17.9 ± 1.54 | -4.52 ± 5.51 | -7.16 ± 8.30 | -5.63 ± 6.61 | NA | 9.79 ± 4.43 | NA | NA | 6.77 ± 4.62 |
| $\beta_{W21}$ | Linear effect of pre-mating liveweight on lamb survival for twins, kg^-1^ | -0.38 ± 0.040 | -0.71 ± 0.070 | 0.18 ± 0.21 | 0.31 ± 0.33 | 0.21 ± 0.27 | NA | -0.34 ± 0.21 | NA | NA | -0.28 ± 0.23 |
| $\beta_{W22}$ | Quadratic effect of pre-mating liveweight on lamb survival for twins, kg^-2^ | 0.0036 ± 0.00043 | 0.0072 ± 0.00079 | -0.0016 ± 0.0020 | -0.0030 ± 0.0032 | -0.0018 ± 0.0028 | NA | 0.0035 ± 0.0024 | NA | NA | 0.0032 ± 0.0027 |
| $\beta_{30}$ | Intercept term for the relationship between age and weight and lamb survival for triplets | 4.66 ± 4.87 | 19.7 ± 9.35 | NA | NA | NA | NA | -1.50 ± 8.54 | NA | NA | -82.8 ± 50.1 |
| $\beta_{W31}$ | Linear effect of pre-mating liveweight on lamb survival for triplets, kg^-1^ | -0.21 ± 0.22 | -0.88 ± 0.41 | NA | NA | NA | NA | 0.066 ± 0.38 | NA | NA | 4.05 ± 2.47 |
| $\beta_{W32}$ | Quadratic effect of pre-mating liveweight on lamb survival for triplets, kg^-2^ | 0.0022 ± 0.0024 | 0.0095 ± 0.0045 | NA | NA | NA | NA | -0.00071 ± 0.0041 | NA | NA | -0.050 ± 0.030 |
| $\beta_{40}$ | Intercept term for the relationship between age and weight and lamb survival for quadruplets | -0.49 ± 44.4 | NA | NA | NA | NA | NA | -21.9 ± 45.8 | NA | NA | NA |
| $\beta_{W41}$ | Linear effect of pre-mating liveweight on lamb survival for quadruplets, kg^-1^ | -0.036 ± 1.97 | NA | NA | NA | NA | NA | 0.95 ± 2.04 | NA | NA | NA |
| $\beta_{W42}$ | Quadratic effect of pre-mating liveweight on lamb survival for quadruplets, kg^-2^ | 0.00078 ± 0.022 | NA | NA | NA | NA | NA | -0.010 ± 0.023 | NA | NA | NA |
|  |  |  |  |  |  |  |  |  |  |  |  |
| $G_{M}$ | Growth rate advantage of ram lamb over ewe lamb, kg day^-1^ | 0.011 ± 0.00091 | 0.010 ± 0.0012 | 0.015 ± 0.0040 | 0.0073 ± 0.0060 | 0.015 ± 0.0039 | NA | 0.015 ± 0.0019 | NA | NA | 0.017 ± 0.0024 |
| $\gamma_{1}$ | Lamb growth rate for birth rank 1, kg day^-1^ | 0.15 ± 0.0044 | 0.16 ± 0.0062 | 0.063 ± 0.023 | 0.080 ± 0.035 | 0.28 ± 0.014 | NA | 0.18 ± 0.016 | NA | NA | 0.21 ± 0.024 |
| $\theta_{1}$ | Effect of pre-mating ewe liveweight on lamb growth rate for birth rank 1, day^-1^ | 0.0022 ± 0.00010 | 0.0018 ± 0.00015 | 0.0038 ± 0.00044 | 0.0046 ± 0.00071 | -0.00042 ± 0.00032 | NA | 0.0019 ± 0.00038 | NA | NA | 0.0016 ± 0.00060 |
| $\gamma_{2}$ | Lamb growth rate for birth rank 2, kg day^-1^ | 0.14 ± 0.0050 | 0.13 ± 0.0064 | 0.087 ± 0.034 | 0.19 ± 0.057 | 0.14 ± 0.024 | NA | 0.15 ± 0.012 | NA | NA | 0.17 ± 0.018 |
| $\theta_{2}$ | Effect of pre-mating ewe liveweight on lamb growth rate for birth rank 2, day^-1^ | 0.0016 ± 0.00012 | 0.0015 ± 0.00015 | 0.0025 ± 0.00063 | 0.0015 ± 0.0011 | 0.0016 ± 0.00052 | NA | 0.0016 ± 0.00028 | NA | NA | 0.0011 ± 0.00046 |
| $\gamma_{3}$ | Lamb growth rate for birth rank 3, kg day^-1^ | 0.18 ± 0.032 | 0.14 ± 0.045 | NA | NA | NA | NA | 0.17 ± 0.042 | NA | NA | 0.25 ± 0.14 |
| $\theta_{3}$ | Effect of pre-mating ewe liveweight on lamb growth rate for birth rank 3, day^-1^ | 0.00067 ± 0.00074 | 0.0011 ± 0.0010 | NA | NA | NA | NA | 0.0012 ± 0.00095 | NA | NA | -0.00074 ± 0.00355 |
| $\gamma_{4}$ | Lamb growth rate for birth rank 4, kg day^-1^ | -0.017 ± 0.19 | NA | NA | NA | NA | NA | -0.016 ± 0.16 | NA | NA | NA |
| $\theta_{4}$ | Effect of pre-mating ewe liveweight on lamb growth rate for birth rank 4, day^-1^ | 0.0049 ± 0.0043 | NA | NA | NA | NA | NA | 0.0049 ± 0.0036 | NA | NA | NA |
| $\sigma_{L}$ | Perturbation amplitude for lamb growth rate, kg day^-1/2^ | 0.76 ± 0.0039 | 0.70 ± 0.0048 | 0.83 ± 0.018 | 1.10 ± 0.028 | 0.80 ± 0.016 | NA | 0.71 ± 0.0088 | NA | NA | 0.62 ± 0.010 |
|  |  |  |  |  |  |  |  |  |  |  |  |

^1^ Parameters reported for ewe lambs (liveweight at 8 months of age was used for liveweight)

^2^ Value reported (±SEM)

^3^ NA indicates that there are not sufficient data to estimate the parameter.

**Supplementary Figure:**

Figure S1. The distribution of pre-mating ewe liveweight for age 2−6 (for all flocks).


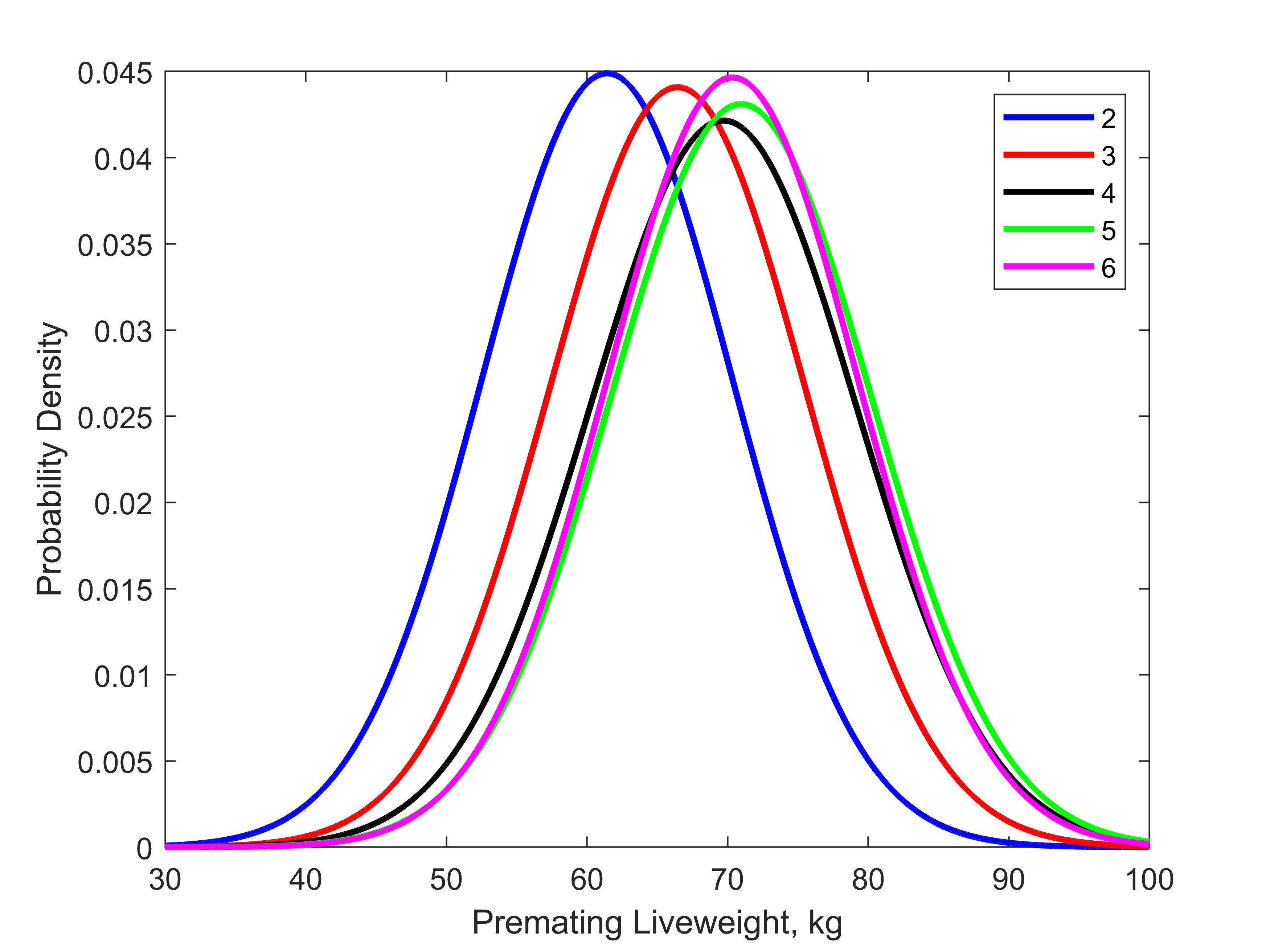


Figure S2. The effect of pre-mating ewe liveweight on lamb growth rate for birth rank 1−4 for all ewes in all flocks. Dotted lines denote SEM.


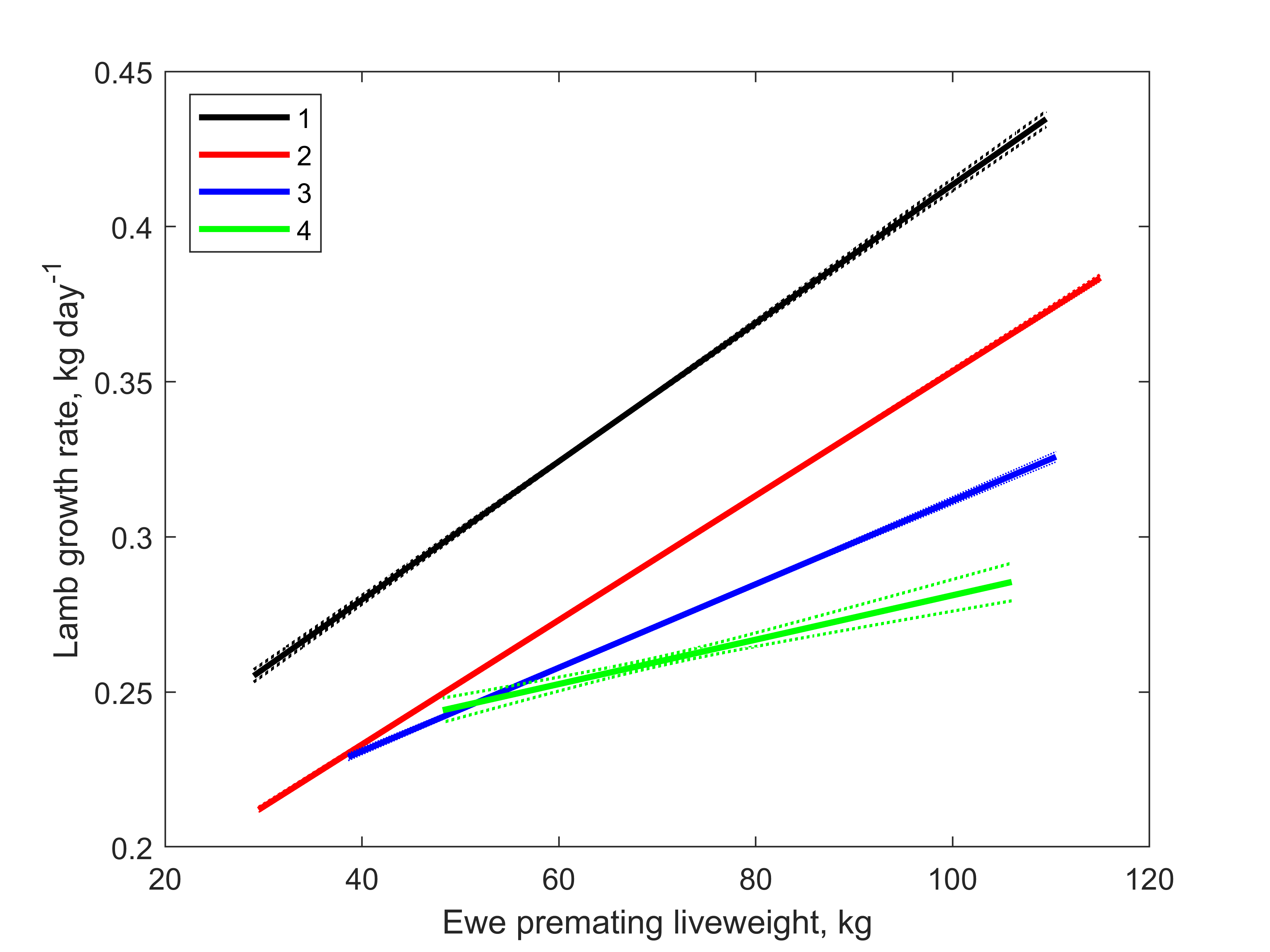


Figure S3. The distribution of pre-mating liveweight (8 months of age) for ewe lambs (for all flocks).


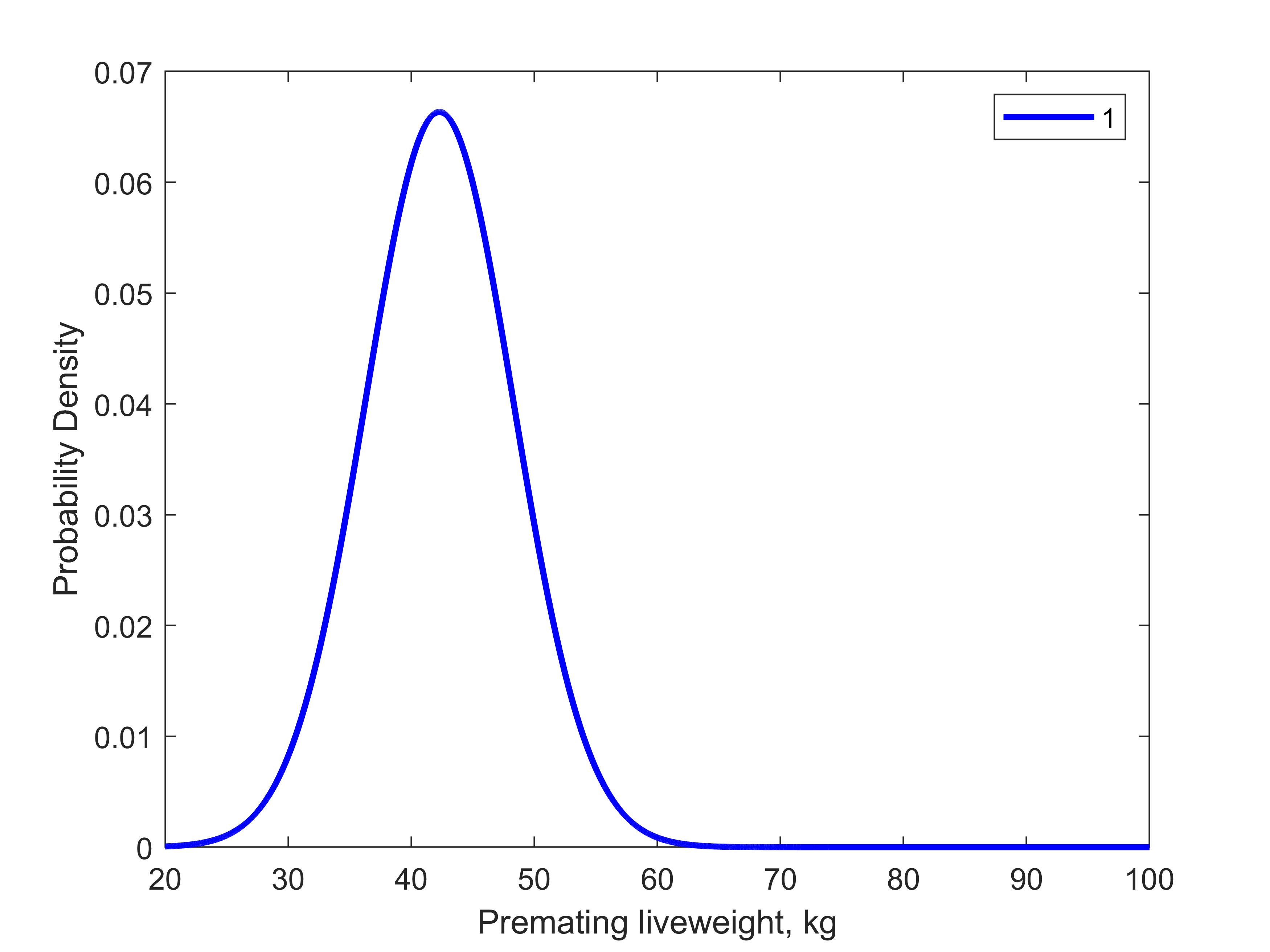


Figure S4. The distribution of the number of fetuses for the Flock 2 (o) and the compound lognormal-binomial model distribution fit (+) to the data (adjusted for weight effects on the number of ova via Eq. 1,2). The mean number of fetuses was 0.72 and the standard deviation in the number of fetuses was 0.71.


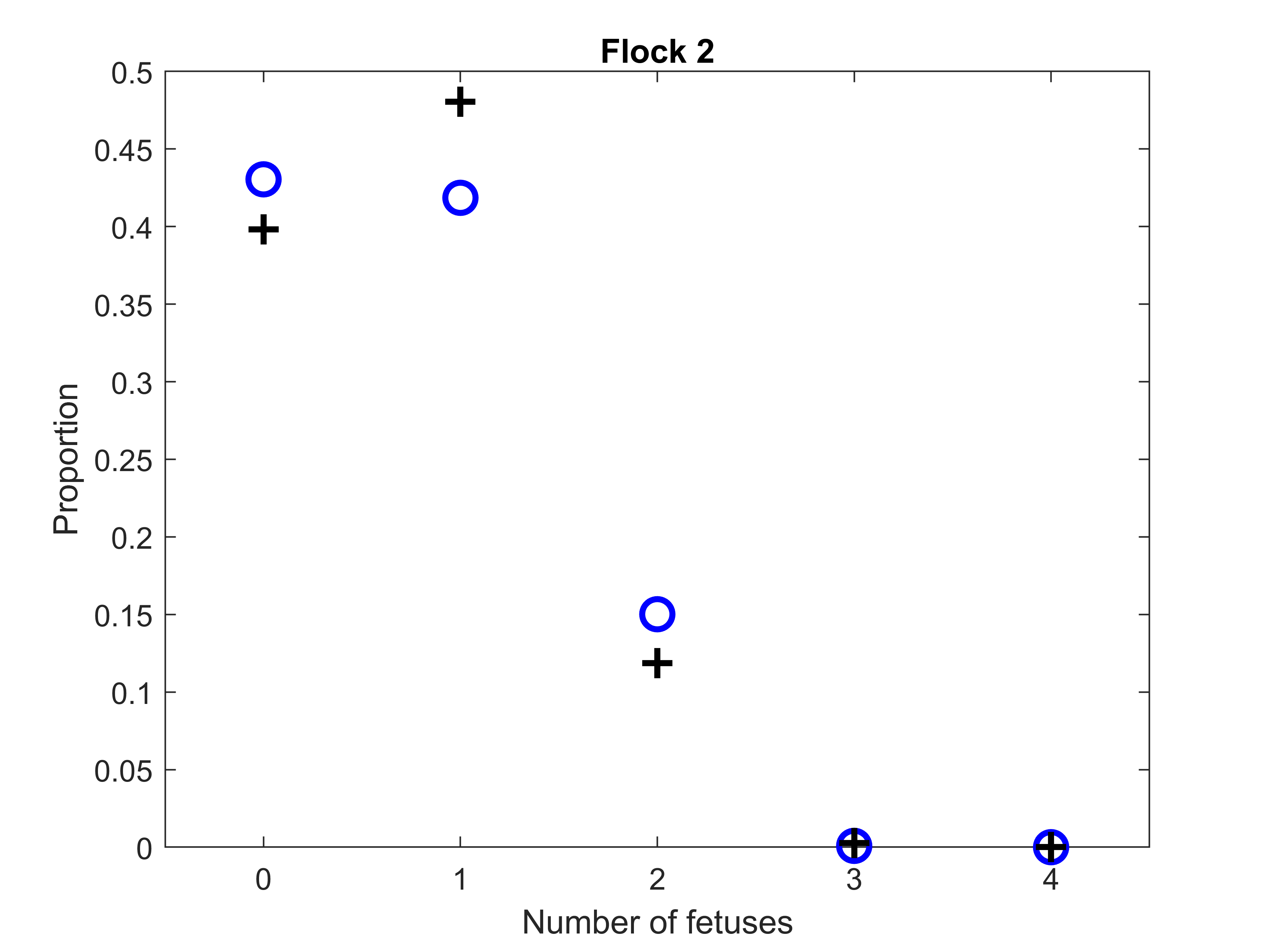


Figure S5. The effect of ewe lamb pre-mating liveweight on the probability of lamb survival for 1−3 fetuses (for all ewe lambs) for all flocks. Dotted lines denote SEM.


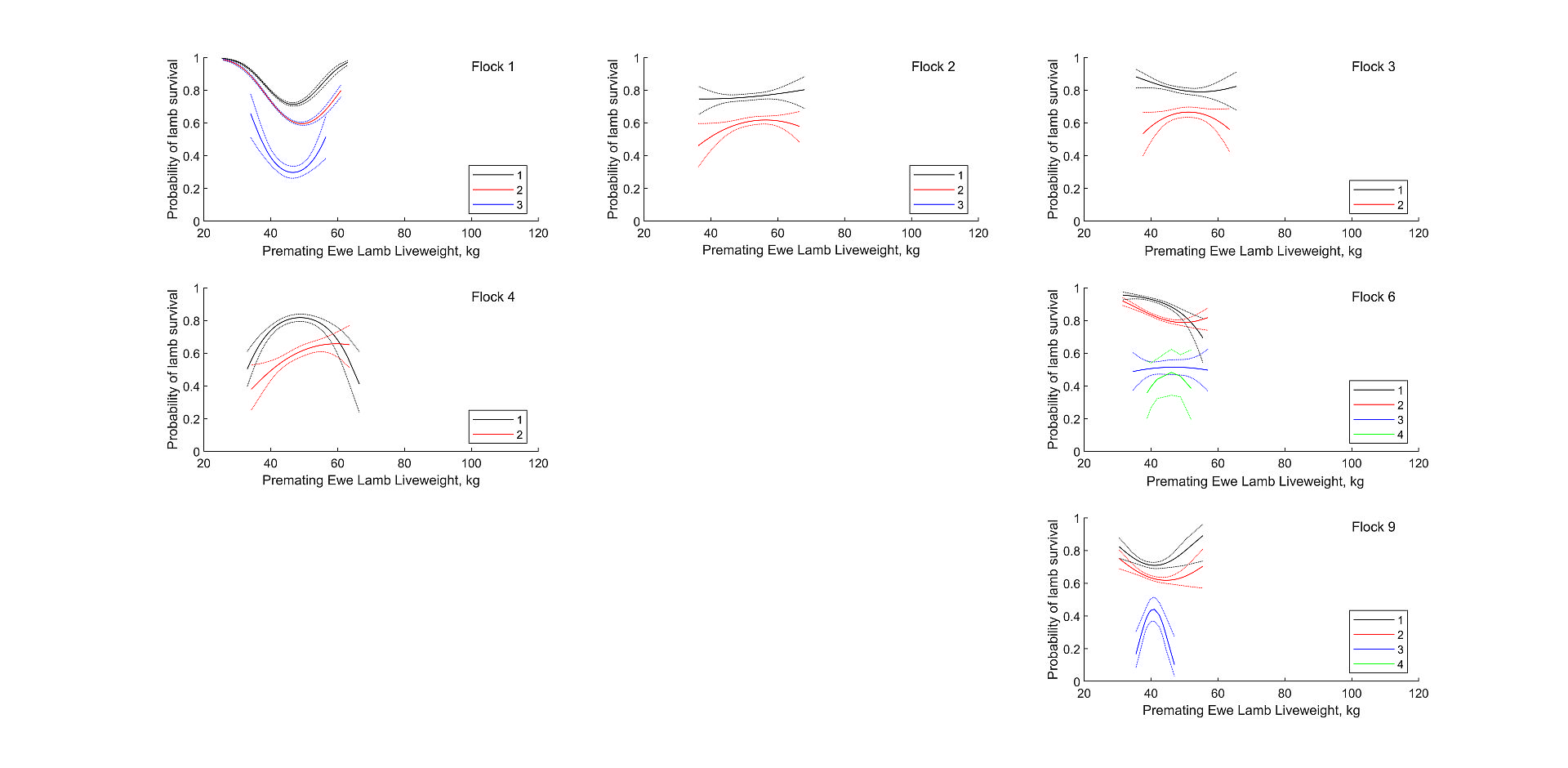


Figure S6. The effect of pre-mating ewe lamb liveweight on lamb growth rate for 1−4 fetuses for each flock (1−9). Dotted lines denote SEM.


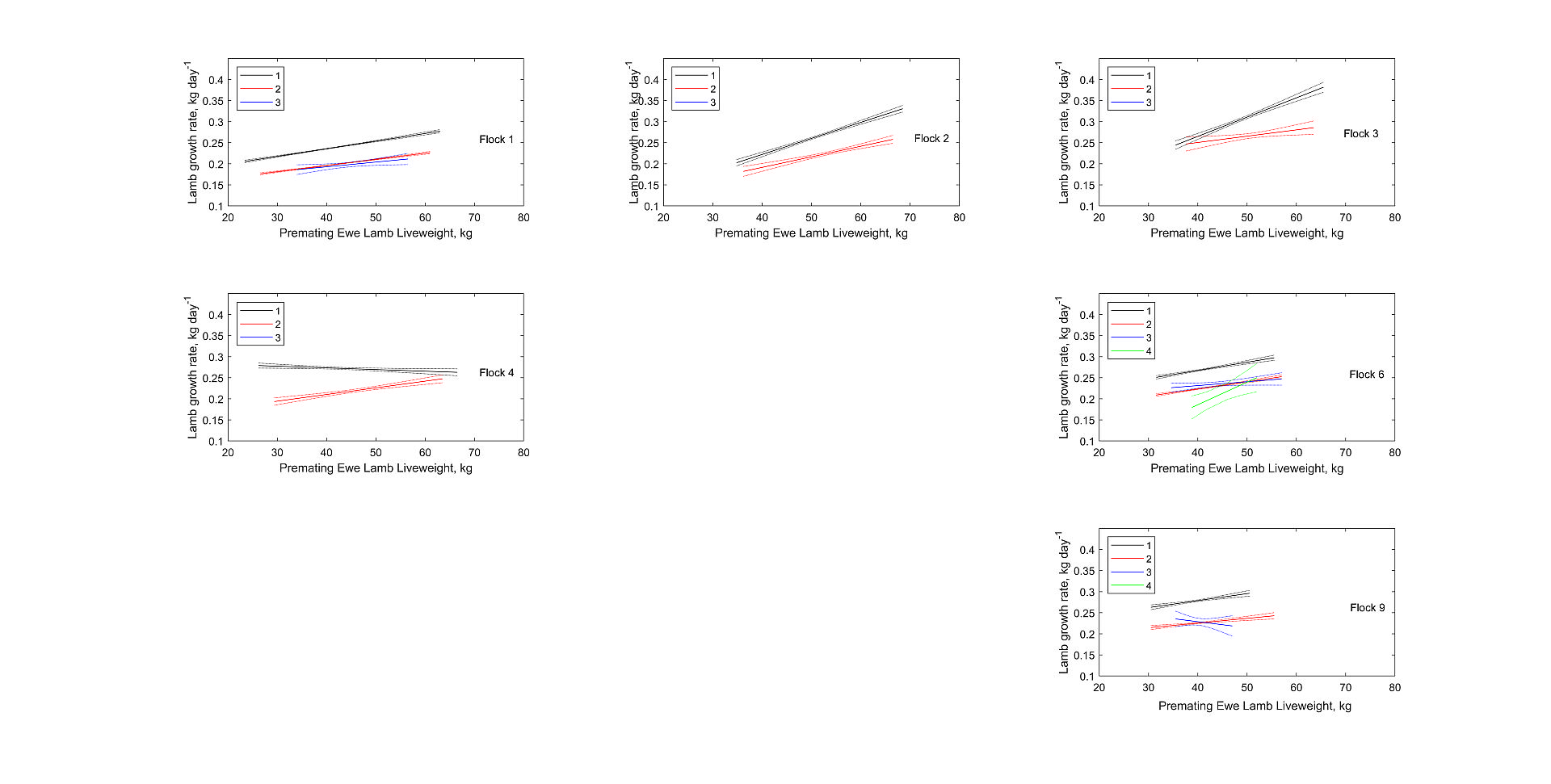


Figure S7. The effect of change in the average pre-mating ewe lamb liveweight on the total weight of lambs weaned per ewe exposed to the ram (for ewe lambs in flocks 1−4, 6, 9) (A). The effect of change in the average ovulation rate on the total weight of lambs weaned per ewe exposed to the ram (B). The effect of change in the standard deviation in ovulation rate on the total weight of lambs weaned per ewe exposed to the ram (C). The effect of change in the embryo/fetal survival probability on the total weight of lambs weaned per ewe exposed to the ram (D). The effect of change in the lamb survival probability on the total weight of lambs weaned per ewe exposed to the ram (E). The effect of change in probability of conception success (*f*) on the total weight of lambs weaned per ewe exposed to the ram (F). Square symbols denote the current flock average.


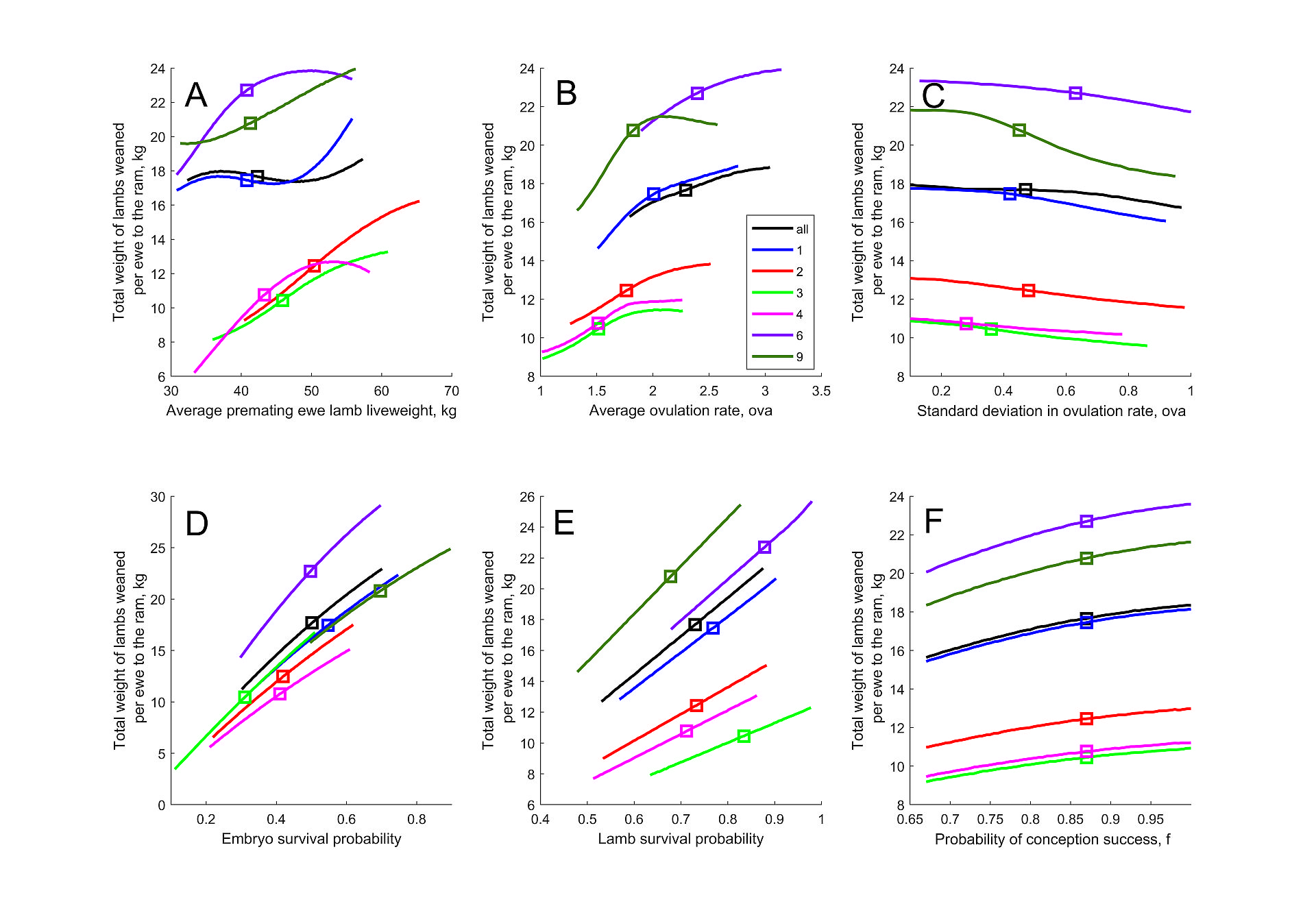

Supplement: txab013_suppl_Supplementary_Materials [file txab013_suppl_supplementary_materials.docx]
